# Integrating Tumor Targeting and Half-Life Extension into a Dual-Surfaced Single-Domain ADAPT Protein

**DOI:** 10.1021/acs.molpharmaceut.6c00683

**Published:** 2026-07-28

**Authors:** Emma Larsson, Moeen Ud-din, Athanasios Bitzios, Enrique Recasens Pérez-Vera, Hanna Tegel, Anna Orlova, Vladimir Tolmachev, Anzhelika Vorobyeva, Sophia Hober

**Affiliations:** a Department of Protein Science, SciLifeLab, 124629 KTH Royal Institute of Technology, Stockholm 106 91, Sweden; b Department of Immunology, Genetics and Pathology, 195558 Uppsala University, Uppsala 751 85, Sweden; c Department of Medicinal Chemistry, 195558 Uppsala University, Uppsala 751 23, Sweden

**Keywords:** protein engineering, ADAPT, tumor targeting, half-life extension, folate receptor alpha, albumin

## Abstract

Folate receptor alpha (FRα) is frequently overexpressed in ovarian cancer while showing limited expression in healthy tissues. Targeting of FRα has been extensively investigated using antibody-based therapies but less so with small scaffold proteins. Small scaffold proteins represent an alternative approach with potential advantages such as site-specific conjugation and deep tumor penetration but are limited by a short half-life. Here, we describe the development of a dual-surfaced ADAPT scaffold protein (6 kDa) with simultaneous bispecificity for FRα and albumin. Using combinatorial libraries and phage display, we identified a candidate, termed ADAPT22, with high isoform selectivity and subnanomolar affinity for FRα (*K*_D_ = 0.8 nM), paired with a low nanomolar affinity for albumin (*K*_D_ = 2.4 nM). Importantly, ADAPT22 is able to interact with both targets simultaneously, truly combining extended retention in blood via albumin association and tumor targeting via FRα within a single-domain protein. The simultaneous nature was verified *in vitro* to soluble proteins and to FRα-positive cells and *in vivo* using a mouse xenograft model. In mice, ADAPT22 demonstrated markedly extended blood retention, reduced renal uptake, and specific accumulation in FRα-positive tumors, confirming simultaneous dual-surface binding. To the best of our knowledge, this is the smallest half-life extended affinity protein designed for therapeutic targeting of FRα.

## Graphical abstract

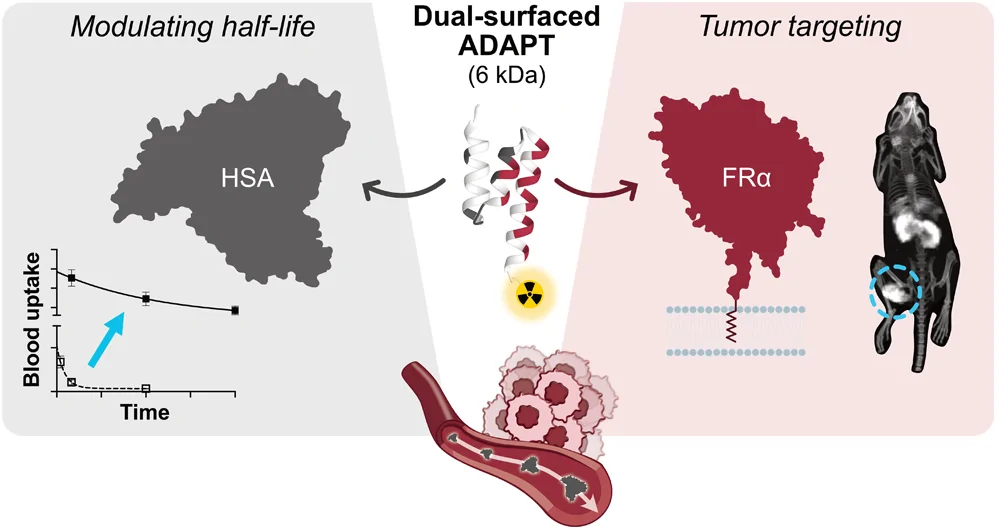

## Introduction

Ovarian cancer originates in the ovary or in the connecting fallopian tube and is associated with nonspecific early symptoms. Consequently, most patients are diagnosed at advanced stages when the cancer has spread within the abdomen or to other distant sites. First-line treatment involves surgery and platinum-based chemotherapy. However, about 75% of advanced-stage patients relapse within two years, and a majority will develop platinum-resistant tumors.^1^ The poor prognosis and limited treatment options for advanced-stage ovarian cancer warrant the development of new targeted therapies.

Folate receptor alpha (FRα) is a GPI-anchored cell surface protein that binds and transports folate into the cell. It has been identified as an attractive target for ovarian cancer, given its widespread expression in up to 90% of ovarian tumors, and limited expression in healthy tissues.^2–5^ Importantly, upregulation of FRα is maintained in platinum-resistant tumors.^6,7^ To date, the only approved FRα-targeted therapy is mirvetuximab soravtansine (Elahere), an antibody-drug conjugate consisting of a humanized IgG1 linked to a cytotoxic payload (DM4). It is approved by the FDA and EMA for platinum-resistant ovarian cancer with high expression of FRα.^8,9^ New FRα-targeting strategies, aiming to broaden the therapeutic landscape to include patients with moderate to low FRα-expression or to improve upon therapeutic efficacy of mirvetuximab soravtansine, are under development. Current research includes new antibody–drug conjugates,^10^ radiolabeled small antibody formats,^11^ albumin-binding radiofolates,^12^ and CAR-T cell therapy.^13^

A less explored alternative for FRα-targeting is the use of non-antibody-based scaffold proteins. Alternative scaffold proteins, such as affibodies, DAPRins, adnectins, and knottins, have been extensively explored for targeting of other tumor-associated receptors, such as HER2, EGFR, EpCAM, and integrins.^14–16^ These alternative scaffold proteins differ in structure and origin but share common features linked to their small size (typically 4–20 kDa) and robust structure. Key advantages include high stability, modularity, and ease of production in bacterial systems or by chemical peptide synthesis. Furthermore, small size can be beneficial in clinical settings by enabling a high tissue extravasation rate and efficient tumor penetration.^17^ However, their small size also leads to rapid renal clearance and short circulatory half-life, limiting the delivery of cytotoxic payload to tumors.^14,18^ Although a short half-life, leading to a low tumor accumulation over time, can be partially mitigated by frequent administration, the high renal uptake still poses a relevant safety concern. This is of particular concern for radionuclide therapy, in which the radiosensitive kidneys are often the dose-limiting organ.^19^

A common strategy for the half-life extension of alternative scaffold proteins is genetic fusion to an albumin-binding protein. By exploiting albumin association, the small fusion proteins can take advantage of the extraordinary half-life of human serum albumin (HSA), which circulates in blood for about 3 weeks. The long circulatory half-life of albumin is attributed to its large size above the renal filtration barrier in combination with its ability to undergo FcRn-mediated recycling.^20^ One of the most extensively studied HSA-binding proteins is the albumin-binding domain (ABD) from streptococcal protein G, a three-helical bundle protein that interacts with an epitope on HSA distinct from the FcRn-binding site.^21–23^ This ABD, along with engineered versions thereof, has been explored as a fusion partner for affibodies,^24,25^ ADAPTs,^26^ DARPins,^27,28^ and rapidly cleared antibody formats,^29^ with reports of dramatic improvements in half-life and drastic reduction in kidney uptake, enabling therapeutic applications.

Although fusion to ABD is a validated strategy for half-life extension of small scaffold proteins, the addition of an ABD fusion partner partially offsets the advantages of minimal size, including compatibility with peptide synthesis. To address this, we have engineered the ABD itself to function as a disease-targeting scaffold protein, called ABD-derived affinity protein (ADAPT).^18^ In the first ADAPT generation, we developed a binder for diagnostic imaging of HER2, called ADAPT6. Due to the position of the HER2-binding surface in helices 1 and 3, interaction with albumin prevented binding of ADAPT6 to HER2.^30^ Thus, to enable *in vivo* targeting, the albumin binding of ADAPT6 was obliterated by several point mutations. In patients, technetium-99-labeled ADAPT6 showed excellent safety, tolerability, and highly specific accumulation in HER2-expressing breast cancer lesions,^31^ supporting the use of the ADAPT scaffold for the development of targeting agents. To enable dual functionality, the scaffold was redesigned by relocating the novel binding surface to helices 1 and 2, opposite the albumin-binding interface in helices 2 and 3, resulting in a dual-surfaced ADAPT library.^32^ This library allows for the development of bispecific ADAPTs, in which half-life extension and the recognition of disease-specific targets are combined within a single-domain protein, merely at a size of 6 kDa. A key feature of this bispecific ADAPT library is its capability to select for proteins that bind HSA and the disease-associated target simultaneously. Simultaneous binding is critical since circulating albumin-associated ADAPT must be able to recognize and bind to the disease target upon encounter, without interference from the albumin interaction. Another important feature of the ADAPT is an intrinsic lack of cysteines, which enables site-specific conjugation to cytotoxins, chelators and other therapeutic moieties. Several bispecific ADAPTs have been successfully generated using this library, including ADAPTs targeting HSA in combination with TNFα, angiogenin, MYDGF, or insulin, and their capacity to simultaneously interact with both targets has been demonstrated *in vitro*.^32^

In this work, we have utilized the bispecific ADAPT library to develop an ADAPT that targets FRα and HSA, thus combining ovarian tumor targeting and albumin-mediated half-life extension within a compact single-domain protein. Phage display selection was employed to identify an initial ADAPT candidate to an appropriate epitope on FRα and further to improve the binding strength by stepwise affinity maturation. Insights into the FRα paratope, gained by deep sequencing and alanine scanning mutagenesis, guided us to expand the binding interface by incorporating a completely new library position, which ultimately allowed for the greatest affinity improvement. However, the new alterations to the paratope resulted in decreased stability, which was resolved by helix stabilization through rational design. The final ADAPT candidate, demonstrating high stability, subnanomolar affinity, and isoform selectivity to FRα, combined with an ability to simultaneously interact with HSA, was radiolabeled to study its biodistribution in mice. The mouse model confirmed a markedly extended blood retention due to albumin association, together with reduced renal uptake and specific accumulation in FRα-positive tumors, supporting the simultaneous binding of this dual-surfaced ADAPT protein.

## Experimental Section

### Design and Construction of Maturation Libraries

The design of the first maturation library was based on deep sequencing data of the naive selection using the amino acid distribution of the best performing sequence cluster (Supporting Information Figure S3) to guide the library randomization. Four positions were locked due to high amino acid conservation (over 90%), while the remaining positions were randomized to varying degrees (Supporting Information Figure S4). Proline, glycine, and cysteine were excluded to avoid structural disruption or disulfide bond formation.

The design of the second maturation library was guided by alanine scanning mutagenesis of ADAPT_FRα_mat1_. Positions identified as important for FRα-binding were softly randomized (50–70% retention of the original residue) and restricted to amino acids of similar biophysical properties (Supporting Information Figure S8). Positions 18 and 31 were locked to alanine. The remaining positions, including a new library position in the N-terminal (previously N7), were fully randomized excluding proline, glycine and cysteine. Both maturation libraries were based on the highly stable scaffold ABD_stable_.^33^

Both maturation libraries were ordered from Ella Biotech (Germany) as a single-stranded oligo covering helices 1 and 2, with library positions diversified via trinucleotide randomization. The nonrandomized helix 3 was synthesized by Integrated DNA Technologies (USA) with a 15 bp overlap with the library oligo. The fragments were assembled by 10 cycles of PCR using Q5 high-fidelity DNA polymerase (NEB), followed by 10 amplification cycles with flanking primers to introduce XhoI and NheI sites for cloning. The assembled libraries were cloned into the phagemid vector pSE^32^ upstream of protein Z and pIII, and electroporated into *E. coli* XL1-Blue (produced in-house), yielding a library size of 5 × 10^8^ and 4 × 10^8^ for maturation library 1 and 2, respectively. Correct assembly was confirmed by colony PCR, Sanger sequencing (Eurofins), and deep sequencing by Illumina MiSeq (National Genomics Infrastructure, SciLifeLab).

### Biotinylation of the Target Protein

The folate receptors were produced as full-length proteins (excluding the propeptide) in CHO cells within the Human Secretome Project, as previously described.^34^ Human FRα was biotinylated using 20× molar excess of EZ-Link Sulfo-NHS-LC-Biotin (Thermo Scientific) in PBS (137 mM NaCl, 2.7 mM KCl, 10 mM Na_2_HPO_4_, 1.8 mM KH_2_PO_4_, pH 7.4) for 30 min at room temperature (RT), followed by removal of excess biotin using a NAP-5 column (Cytiva). Successful biotinylation was verified in a binding test to streptavidin-coated Dynabeads M-280 (Thermo Scientific) with detection by SDS–PAGE.

### Phage Display Selections

Phage display selections against human FRα (Supporting Information Table S1) were carried out using a previously described semiautomated procedure,^35^ with automated library panning and plate-based phage amplification. In brief, libraries precleared against streptavidin-coated beads were incubated with biotinylated FRα in solution, prior to capture on streptavidin-coated Dynabeads M-280 (Thermo Scientific). The beads were washed in PBS-T (PBS with 0.1% Tween20), and bound phages were eluted with 50 mM glycine (pH 2.2) for 10 min. The eluted phages were neutralized with an equal volume of 100 mM Tris-HCl in PBS (pH 8.0) and amplified in *E. coli* XL1-Blue for subsequent rounds of panning.

The naive selection used a previously described bispecific ADAPT library^32^ and was carried out in four rounds with decreasing FRα concentration (150 nM to 25 nM) and increasing number of washes (2× to 12× washes) at RT (Supporting Information Table S2a).

The first affinity maturation was performed in four rounds with maturation library 1 (Supporting Information Table S2b). Round 1 applied gentle conditions (100 nM FRα, RT, 4 washes). Rounds 2 and 3 applied increased stringency through reduced FRα concentration (25 nM and 5 nM FRα), elevated temperature (37 °C), and increased washing (15× and 10×). Additionally, round 3 applied off-rate selection using 250× molar excess of nonbiotinylated FRα during extended washing (2 h at 37 °C or 18 h at RT). Round 4 served as a recovery round (50 nM FRα, RT, 9× washes).

The second affinity maturation was carried out in three rounds with maturation library 2, with all steps at 37 °C (Supporting Information Table S2c). Round 1 used 50 nM FRα with 5x washes. Round 2 applied increased stringency (5 nM FRα, 10× washes) and included an extended off-rate wash with 500× molar excess of nonbiotinylated FRα (20 h at RT or 4 h at 37 °C). In round 3, the input was split into two tracks, one with 5 nM FRα and a more rigorous washing at increased speed (2000 rpm), and the other with 1 nM FRα and an additional stringent off-rate wash with 500× molar excess of nonbiotinylated FRα (20 h at RT, or 3 h at 37 °C followed by 17 h at RT).

### Deep Sequencing and Data Analysis

The phagemid output from each selection round was isolated from infected XL1-Blue *E. coli* cells using a QIAprep Spin Miniprep Kit (Qiagen). The extracted phagemids (50 ng) were amplified by 15 cycles of PCR using primers containing Illumina adapters and unique sample barcodes. The amplified fragments were purified by gel extraction (2% GTG agarose, QIAquick Gel Extraction Kit (Qiagen)), pooled in equal amounts, and submitted to the National Genomics Infrastructure (Stockholm, Sweden) for deep sequencing using an Illumina MiSeq v2.

The sequencing data (FASTQ files) were analyzed using PipeBio (pipebio.com). Paired-end reads were merged and mapped to the ADAPT variable region, followed by clustering (100% identity) of unique sequences, MAFFT alignment of the most enriched sequences, and phylogenetic analysis. Hit selection was guided by phylogenetic clustering, abundance, and enrichment trends across selection rounds.

### Subcloning of ADAPT Candidates from the Selections

Deep sequencing data guided the selection of 12 enriched clones from the naive panning and 24 clones from the first affinity maturation for subcloning and production as soluble proteins. Clones were rescued from the selection outputs by a previously described PCR-based method,^32^ using clone-specific oligos covering the library positions in helix 1 and 2 and introducing restriction sites for cloning. The rescued fragments were subcloned into a carbenicillin-resistant, T7 inducible expression vector carrying the constant helix 3, to assemble the full-length ADAPT sequence.

### Site-Directed Mutagenesis for Single Amino Acid Substitutions

Alanine scanning mutagenesis was performed on the 11 library positions in ADAPT_FRα_mat1_ to identify critical residues for FRα binding. Each library position was individually substituted to alanine, or to valine for an already existing alanine (position 18). Primers were designed to introduce the substitution directly into the expression vector by 16 cycles of PCR using Phusion high-fidelity DNA polymerase (Thermo Scientific), followed by DpnI (NEB) digestion of the parental plasmid and transformation into chemically competent *E. coli* Top10. Successful mutagenesis was verified by Sanger sequencing (Eurofins).

Residue S8 in ADAPT_FRα_mat2_ was subjected to site-directed mutagenesis to stabilize the N-capping motif in helix 1. Position 8 was identified as the N3 position within the N-capping motif, according to SWISS-MODEL homology modeling^36,37^ using the wild-type ABD (PDB 1GJT) as template. Four substitutions (S8Q, S8N, S8E, S8D) were selected based on amino acid preference in the N3 position of naturally occurring alpha helices.^38–40^ The substitutions were introduced using the same procedure as described above.

### Production and Purification of ADAPT Variants

Expression vectors with sequence-confirmed ADAPT inserts were transformed by heat-shock into *E. coli* BL21­(DE3) for protein expression. Cells were cultured in 100 mL TSB-Y (tryptic soy broth with 5 g/L yeast extract) with 100 μg/mL carbenicillin at 37 °C, 150 rpm until the point of exponential growth (OD_600_ 0.5–1), at which 1 mM IPTG was added, followed by overnight incubation at 25 °C, 150 rpm. The cells were lysed by sonication and the proteins were purified by immobilized metal ion affinity chromatography (IMAC) or on HSA-Sepharose. For IMAC, the clarified cell lysates were loaded onto equilibrated gravity flow columns packed with 2 mL HisPur cobalt resin (Thermo Scientific), followed by washing with 30 mL IMAC wash buffer (50 mM sodium phosphate, 300 mM NaCl, 30 mM imidazole, pH 7.5). The proteins were eluted with IMAC elution buffer (50 mM sodium phosphate, 300 mM NaCl, 300 mM imidazole, pH 7.5), followed by buffer exchanged to 20 mM NH_4_Ac (pH 9) using PD-10 desalting columns (Cytiva). For HSA-based purification, the clarified cell lysates were loaded onto gravity flow columns packed with 3 mL HSA-Sepharose (coupled in-house). The columns were washed with 30 mL of TST (25 mM Tris, 1 mM EDTA, 0.2 M NaCl, 0.05% Tween20, pH 8) and 6 mL of 5 mM NH_4_Ac (pH 6), followed by elution with 0.5 M acetic acid (pH 2.8). The protein samples were freeze-dried and resuspended in PBS (pH 7.4). The protein concentration was determined by Qubit 4 fluorometer (Invitrogen). High purity was verified by SDS–PAGE and MALDI TOF/TOF mass spectrometry.

Proteins for radiolabeling were further purified on semipreparative RP-HPLC (Zorbax 300SB-C18 column, 9.4 mm × 250 mm, particle size 5 μm; Agilent). Proteins were eluted using a gradient of 30–60% B over 35 min at a flow rate of 3 mL/min (A, 0.1% trifluoroacetic acid in water; B, 0.1% trifluoroacetic acid in acetonitrile).

### Pool Cloning of Selection Output for Kinetic Screening

After the final round of the second affinity maturation, phagemid outputs from all four tracks were allowed to infect XL1-Blue *E. coli* cells. The phagemid pool from each track was isolated using a QIAprep Spin Miniprep Kit (Qiagen). Cloning of the each ADAPT gene pool (10 ng template) was performed by introducing restriction sites via flanking primers in 15 cycles of PCR using Q5 high-fidelity DNA polymerase (NEB), followed by ligation into a bacterial expression vector containing an N-terminal 6xHis-tag, an inducible T7 promoter and carbenicillin resistance, and chemical transformation into *E. coli* BL21­(DE3). Colony PCR confirmed insert presence and clones with expected size were selected for screening. Single colonies were grown overnight in 96-well plates at 37 °C, 220 rpm. Cultures were expanded into 4 mL of TSB-Y with 100 μg/mL carbenicillin in 24-well plates and grown in 37 °C, 150 rpm until OD_600_ > 0.8, at which 1 mM IPTG was added followed by incubation overnight at 25 °C, 150 rpm. Cells were harvested by centrifugation (3500*g* for 20 min), lysed in 0.5 mL lysis buffer (0.5 mg/mL lysozyme (Sigma-Aldrich), 8 U/ml DNase I (Sigma-Aldrich), 1 mM MgCl_2_ in 20 mM Tris-HCl, pH 8) for 1 h at RT, and clarified by centrifugation (16.000*g* for 20 min at 4 °C). Clarified cell lysates, containing individual ADAPT variants (332 in total), were screened in BLI against 1000 nM FRα, as described below.

### Biolayer Interferometry (BLI)

Kinetic screening was performed by BLI using an Octet RED96 (Pall ForteBio). ADAPT variants from the affinity maturations, and the alanine scan mutagenesis, were expressed in *E. coli* BL21­(DE3) and screened directly from cell lysates. Octet HIS1K biosensors (Sartorius) were dipped into cell lysates for 300–600 s to load the ADAPT variants via their N-terminal 6xHis-tag, and washed in PBS-T (PBS, 0.05% Tween20, pH 7.4). Baseline was recorded in PBS-T, followed by association to FRα (1000 nM in PBS-T) for 250 or 300 s, and dissociation in PBS-T for 500 or 600 s. The biosensors were regenerated with 10 mM glycine-HCl (pH 1.5)/PBS-T for 3× 10 s/10 s. All steps were performed at 25 °C with 1000 rpm shaking. A reference biosensor without ADAPT was included for background subtraction. The data were processed by reference subtraction, interstep correction, and Savitzky–Golay filtering in Data Analysis 10.0 (ForteBio).

### Surface Plasmon Resonance (SPR)

A Biacore T200 (Cytiva) was used to determine the affinity to FRα and HSA, assess simultaneous binding, and perform epitope binning on FRα. FRα and HSA were immobilized on separate flow cells of a Series S sensor chip CM5 (Cytiva) via amine coupling, according to the manufacturer’s instructions, with an empty reference flow cell for background subtraction. All experiments were performed at 25 °C with PBS-T (PBS, 0.05% Tween20, pH 7.4) as running buffer and sample buffer.

Affinity (*K*_D_) to immobilized FRα and HSA was determined by injecting ADAPT variants in 2-fold serial dilutions at 30 μL/min for 250–300 s, followed by dissociation in PBS-T for 600 s (or 1800 s for ADAPT_FRα_v2_ over HSA). Measurements were performed in a multicycle format with surface regeneration using 10 mM HCl for 30 s. Data were reference- and buffer-subtracted in the Biacore Insight Evaluation software, and kinetic constants were determined using a 1:1 binding model.

Simultaneous binding of ADAPT22 (or ADAPT_FRα_v2_) was assessed using a tandem assay. First, 25 nM (or 250 nM) of the ADAPT variant was injected over immobilized HSA for 300 s (or 120 s) at 10 μL/min, immediately followed by an injection of 25–100 nM FRα (or 250 nM) for 200 s (or 120 s) at 30 μL/min to detect a second binding event. As a control, a first injection with buffer followed by a second injection with FRα confirmed no target binding in absence of ADAPT.

Epitope binning analysis of ADAPT22 to FRα was performed in two experiments. First, competition with the natural ligand folate was assessed by injecting a saturating concentration of 5-methyltetrahydrofolic acid (2000 nM; MedChemExpress) over immobilized FRα for 180 s at 10 μL/min, followed by ADAPT22 (100 nM) for 180 s at 30 μL/min. The binding signal of ADAPT22 was compared to buffer control (no folate present). In a second experiment, competition with the antibody moiety of the clinically approved mirvetuximab soravtansine was assessed. Mirvetuximab (M9346A, MedChemExpress) was captured on immobilized FRα at a saturating concentration (500 nM) for 120 s at 10 μL/min, followed by washing with PBS-T and injection of ADAPT22 (100 nM) for 300 s at 30 μL/min. The experiment was repeated in reverse direction, by injecting ADAPT22 (500 nM) followed by mirvetuximab (100 nM).

Cross-reactivity to all human folate receptor isoforms was assessed using a Biacore 8K (Cytiva). Streptavidin was immobilized using amine coupling on flow cell 1 and 2 of a Series S sensor chip CM5 (Cytiva). ADAPT22 was produced with a C-terminal cysteine for site-specific conjugation of biotin (described below). Biotinylated ADAPT22 (100 nM) was captured on flow cell 2 of the streptavidin chip for 180 s at 10 μL/min, while flow cell 1 was served as reference. FRα, FRβ, FRγ, and FRδ (100 nM) were injected sequentially at 30 μL/min (association for 200 s, dissociation for 600 s in PBS-T), with regeneration between samples using 10 mM HCl. Data were double-referenced by subtracting buffer signals and the reference flow cell.

### Circular Dichroism Spectroscopy (CD)

Thermal stability of the ADAPT variants was analyzed using a Chirascan CD spectrometer (Applied Photophysics). All measurements were performed using a 1 mm cuvette and a protein concentration of 0.2 mg/mL in PBS. First, secondary structure was assessed at 20 °C, by recording ellipticity from 195 to 260 nm. Second, melting curves were obtained by monitoring the ellipticity at 221 nm while heating from 5 to 90 °C (5 °C/min). The data were normalized (0 = unfolded, 1 = folded) and fitted to a Boltzmann sigmoidal function to calculate the melting temperature, *T*_m_ (GraphPad Prism version 10). Finally, ability to refold was evaluated by lowering the temperature back to 20 °C and repeating the spectral measurement.

### Analytical Size Exclusion Chromatography (SEC)

The tendency of the ADAPT variants to aggregate was assessed by SEC using a Superdex 75 Increase 5/150 GL column (Cytiva) on a Bio-Rad NGC chromatography system (Bio-Rad). The column was equilibrated with PBS, followed by injection with 25 μL of ADAPT sample (0.2–0.4 mg/mL in PBS) and elution with PBS at a flow rate of 0.45 mL/min. The elution profiles were compared to a calibrant (conalbumin 75 kDa, ovalbumin 44 kDa, carbonic anhydrase 29 kDa, ribonuclease A 13.7 kDa, aprotinin 6.5 kDa) to estimate the molecular size.

Simultaneous binding of ADAPT22 to FRα and HSA was assessed by SEC by the formation of a ternary complex in solution. ADAPT22, FRα, and HSA were mixed at equimolar ratios (10 μM) and injected (25 μL) onto a Superdex 200 Increase 5/150 GL column (Cytiva), followed by elution in PBS at 0.45 mL/min. The resulting chromatogram was compared to chromatograms of only two interacting proteins: 10 μM of ADAPT22 and HSA, or 10 μM of ADAPT22 and FRα.

### Cell Culture

Human cancer cell lines SKOV-3 (ovarian adenocarcinoma), MCF-7 (breast adenocarcinoma), BxPC-3 (pancreatic adenocarcinoma), PC-3 (prostate adenocarcinoma), Ramos (Burkitt’s lymphoma), and Jurkat (acute T cell leukemia) were obtained from ATCC (USA). SKOV-3 was cultured in DMEM (low glucose, Gibco) with 2 mM l-glutamine, 10% fetal bovine serum (FBS; Gibco), and 1% penicillin–streptomycin. MCF-7 was grown in DMEM (Gibco) with 10% FBS and 1% penicillin–streptomycin. BxPC-3, PC-3, Ramos, and Jurkat were cultured in RPMI-1640 with 10% FBS and 1% penicillin–streptomycin. IGROV-1 (ovarian adenocarcinoma) was obtained from Sigma-Aldrich (Merck KGaA, Darmstadt, Germany; catalog no. SCC203) and were cultured in Dulbecco’s modified Eagle medium (DMEM; Gibco, Thermo Fisher Scientific, Waltham, USA) containing 10% FBS, 1% penicillin–streptomycin. All cell lines were grown at 37 °C and 5% CO_2_.

### Biotinylation of ADAPTs for SPR and Flow Cytometry

ADAPT22 was produced with a unique C-terminal cysteine. The protein was reduced with 5 mM TCEP for 30 min at RT, followed by site-specific biotinylation using 20x molar excess of EZ-Link Maleimide-PEG2-Biotin (Thermo Scientific) for 40 min at RT, followed by 3 h on ice. Excess biotin was removed by buffer exchange using a NAP-5 column (Cytiva). A high degree of labeling (>80%) was confirmed by mass spectrometry (4800 MALDI TOF/TOF, Applied Biosystems).

### Flow Cytometric Analysis

The binding specificity of ADAPT22 was evaluated to different cancer cell lines with varying expression of FRα (IGROV-1, SKOV-3, MCF-7, BxPC-3, Ramos, Jurkat). Cells were detached using TrypLE Express Enzyme (Gibco) and resuspended in PBS-B (PBS, 0.5% bovine serum albumin). Cells (100.000 per sample) were collected in a 96-well plate and washed twice with PBS-B by centrifugation (600*g*, 4 °C, 2 min). Cells were incubated with biotinylated ADAPT (5 nM or 10 nM) or PE-conjugated anti-FRα antibody (diluted 1:100; FAB5646P, Bio-Techne) for 40 min at RT with gentle shaking. ADAPT-treated cells were washed once with PBS-B and incubated with 100 μL of PE-conjugated streptavidin (diluted 1:500; Invitrogen) for 30 min on ice. All cells were washed twice with PBS-B and analyzed using a CytoFLEX S Flow Cytometer (laser 561 nm; Beckman Coulter) with detection at 585/42 nm.

The on-cell affinity of ADAPT22 was determined to endogenously expressed FRα on SKOV-3 cells. Biotinylated ADAPT22 was prepared in triplicates in a 2-fold serial dilution (0.156 nM to 320 nM) in either the absence or presence of 20× molar excess of HSA. The HSA-containing samples were preincubated with ADAPT for 2 h at RT to allow complex formation. Cells were harvested and washed as described above. Cells (70.000 per sample) were incubated with the ADAPT samples for 1 h at RT with gentle shaking, washed once in PBS-B, and incubated with streptavidin-PE (diluted 1:500; Invitrogen) for 30 min on ice. After two washes with PBS-B, the mean fluorescent intensity was detected by flow cytometry using a CytoFLEX S, as described above. Data were fitted to a Sigmoidal 4PL function (GraphPad Prism version 10). BxPC-3 cells were included as a negative control, confirming no binding for any of the ADAPT samples. An anti-FRα antibody verified expression of FRα on SKOV-3, with no detectable signal to BxPC-3.

### Radiolabeling and Stability Evaluation

Radiolabeling of ADAPT22 and ADAPT6 with technetium-99m tricarbonyl [^99m^Tc]­Tc­(CO)_3_ was performed site-specifically at the N-terminal hexahistidine tag acting as a chelator,^41^ following an established protocol.^42^ Briefly, technetium-99m pertechnetate was added to a CRS kit (Paul Scherrer Institute, Villigen, Switzerland) and incubated at 100 °C for 30 min to generate the tricarbonyl precursor. The pH was adjusted to 8 with 0.1 M HCl, and the technetium-99m tricarbonyl (30–300 μL, 60–400 MBq) was added to ADAPT22 or ADAPT6 (30 μg, 15–22 μL). Reactions were incubated at 50 °C for 60 min. To remove loosely coordinated ^99m^Tc, histidine (16 μL, 20 mg/mL, 500-fold molar excess) was added and incubated for 10 min at 50 °C. Radiolabeled proteins were purified using NAP-5 size-exclusion columns (Cytiva) pre-equilibrated in PBS. Radiochemical yield (RCY) and radiochemical purity (RCP) were assessed using instant thin-layer chromatography (iTLC) in PBS. Radioactivity was quantified using a Cyclone storage phosphor system (CR 35 BIO Plus, Dürr Medical) and AIDA image analyzer (Elysia-Raytest); activity measurements were performed using an IBC dose calibrator (Comercer, Italy). To evaluate label stability, purified [^99m^Tc]­Tc­(CO)_3_-ADAPT22 (1.5 μg, 60 μL) was incubated with a 500-fold molar excess of histidine (challenge condition) or with PBS (control) in a total volume of 71 μL at 37 °C for 3 h (*n* = 2). Postincubation RCP was normalized to the initial RCP.

### Binding Specificity and Cellular Internalization of [^99m^Tc]­Tc­(CO)_3_-ADAPT22

Specific binding of [^99m^Tc]­Tc­(CO)_3_-ADAPT22 to IGROV-1, SKOV-3, and PC-3 cells was evaluated as previously described.^42^ For blocking, unlabeled ADAPT22 (400 nM) was added to saturate FRα, while control wells received medium alone. After incubation at 37 °C for 30 min, [^99m^Tc]­Tc­(CO)_3_-ADAPT22 (4 nM final) was added, and cells were incubated at room temperature for 4 h. Media were collected, cells were washed, detached with trypsin, and counted. Cell-associated radioactivity was measured using a γ-counter with a 3 in. NaI­(Tl) detector (2480 Wizard, Wallac, Finland). Results were expressed as activity per 1 × 10^6^ cells (*n* = 3).

Internalization and cellular processing in IGROV-1 cells were studied using an acid-wash protocol.^42,43^ Cells were incubated with [^99m^Tc]­Tc­(CO)_3_-ADAPT22 (4 nM) at 37 °C for 1, 2, 4, 6, or 24 h. At each time point, non-cell-associated, membrane-bound, and internalized fractions were collected. Radioactivity in each fraction was quantified using a γ-spectrometer. Internalization efficiency was evaluated by comparing samples incubated at 37 °C with parallel samples maintained on ice (0 °C) to inhibit active uptake. Experiments were performed with or without 100 nM HSA. Data represent mean values of three samples and are normalized to the maximal cell-associated activity at 24 h.

### Animal Studies

The animal experiments were planned and carried out in compliance with the Swedish national legislation concerning the protection of laboratory animals. The experiments were approved by the Ethics Committee for Animal Research at Uppsala University (Permit 5.8.18-00473/2021).

### Biodistribution in NMRI Mice

Biodistribution studies of [^99m^Tc]­Tc­(CO)_3_-ADAPT22 and [^99m^Tc]­Tc­(CO)_3_-ADAPT6 were performed in female NMRI mice (Scanbur A/S, Denmark). The age of the NMRI mice was 8 weeks, and the average weight was 27.2 ± 1.7 g at the time of experiment. Mice (*n* = 4 per group) were intravenously (i.v.) injected with radiolabeled ADAPTs (140 pmol, 1 μg in 100 μL of 1% BSA in PBS). Injected radioactivities were 100 KBq for the 1 and 4 h time points, 1.4 MBq for the 24 h time point, and 10 MBq for the 48-h time point. At 1, 4, 24, or 48 h postinjection (p.i.), mice were euthanized, blood and organs were collected, weighed, and measured for radioactivity using an automatic gamma counter. Organ uptake was expressed as percentage of injected dose per gram (%ID/g), except the gastrointestinal tract and carcass, which were expressed as %ID. All measured values were decay-corrected for physical decay of the radionuclide. Blood retention data were fitted using a one-phase exponential decay model with the plateau constrained to zero in GraphPad Prism (version 10; GraphPad Software, USA). The resulting half-life estimates should be regarded as approximate because they are based on a limited number of time points obtained as part of the biodistribution studies.

### Biodistribution in Tumor-Bearing BALB/c nu/nu Mice

The tumor-targeting properties of [^99m^Tc]­Tc­(CO)_3_-ADAPT22 were evaluated in female BALB/c nu/nu mice (Charles River, Germany) bearing FRα-positive IGROV-1 ovarian carcinoma xenografts or FRα-negative PC-3 prostate cancer xenografts. Xenografts were established by subcutaneous implantation of 1 × 10^7^ of IGROV-1 cells (right hind leg), or 1 × 10^7^ of PC-3 cells (left hind leg) in 100 μL of medium. Experiments were conducted when tumors were clearly visible and reached approximately 50–100 mm^3^; *n* = 3–5 mice per group. The age of the BALB/c nu/nu mice was 12–13 weeks, and the average weight was 18.0 ± 1.5 g at the time of experiment. The average IGROV-1 tumor weight was 0.118 ± 0.093 g (*n* = 12, 0.030–0.240 g), and the average PC-3 tumor weight was 0.055 ± 0.051 g (*n* = 4, 0.009–0.120 g) at dissection. Mice were injected i.v. with [^99m^Tc]­Tc­(CO)_3_-ADAPT22 (140 pmol, 1 μg, 100 μL of 1% BSA in PBS), with the injected radioactivities as described above. For receptor-blocking controls, nonlabeled ADAPT22 (1 mg/mouse) was administered subcutaneously 18 h prior to radiolabeled ADAPT22 injection, and biodistribution was assessed at 24 h p.i. At 4, 24, or 48 h p.i., mice were euthanized, organs and blood were collected and weighed, and radioactivity was measured as described above.

### Micro-SPECT/CT Imaging

Micro-SPECT/CT imaging was performed to obtain a visual confirmation of *ex vivo* biodistribution findings. One IGROV-1-bearing mouse and one PC-3-bearing mouse received intravenous injections of [^99m^Tc]­Tc­(CO)_3_-ADAPT22 (2 μg in 100 μL of 1% BSA in PBS; 20.8 MBq for IGROV-1, 20.6 MBq for PC-3). For the blocking control, an IGROV-1–bearing mouse was administered nonlabeled ADAPT22 (1 mg, s.c.) 18 h prior to injection of radiolabeled ADAPT22 (2 μg, 20.2 MBq). Mice were sacrificed at 24 h p.i., and whole-body SPECT/CT scans were acquired using a nanoScan SPECT/CT system (Mediso Medical Imaging Systems, Hungary). SPECT data were reconstructed using Tera-Tomo 3D software, and CT images were reconstructed using filtered back-projection in Nucline 2.03 software. Fused SPECT/CT images were generated in Nucline 2.03 and displayed as maximum-intensity projections.

## Results

### Generation of the First FRα- and HSA-Binding ADAPT Candidates

With the aim of developing an ADAPT that targets FRα for cancer treatment and albumin for half-life extension, we employed a previously established bispecific ADAPT library.^32^ This combinatorial library has been designed by randomization of 11 positions in helices 1 and 2 to introduce a novel binding surface, while the binding surface to albumin in helices 2 and 3 has been left intact (Figure 1a,c). The library was used in phage display to select binders against recombinant human FRα. The selection output was analyzed by deep sequencing, and the most abundant sequences were clustered based on sequence similarity (Supporting Information Figure S1). A selection of 12 clones from four different sequence clusters were produced and purified for initial characterization. Half of the clones were shown to bind FRα, while all showed retained binding to HSA. Interestingly, the FRα-binding clones were originating from two sequence clusters (Supporting Information Figure S1, green and purple), with only one cluster showing an ability to interact with FRα and HSA simultaneously (purple). Further, the simultaneous bispecific binders displayed desirable biophysical properties, with high thermal stabilities, melting temperatures over 50 °C and no aggregation tendencies, as well as affinities to FRα in the nanomolar range, exemplified by the clone ADAPT_FRα_v2_ in Supporting Information Figure S2. The affinity (*K*_D_) of ADAPT_FRα_v2_ to FRα was determined to be 79 nM, as assessed by surface plasmon resonance (SPR) (Figure 1d, Table 1). Additionally, no binding was detected to homologous FRβ (Supporting Information Figure S2c), a human receptor isoform that shares around 80% sequence identity with FRα.^44^ Further characterization of ADAPT_FRα_v2_ confirmed specific binding to endogenously expressed FRα on the ovarian cancer cell line SKOV-3 (Supporting Information Figure S2h,i). Although the novel binders showed moderate affinity, their rapid dissociation required further optimization to improve kinetic properties.

**Figure 1:**
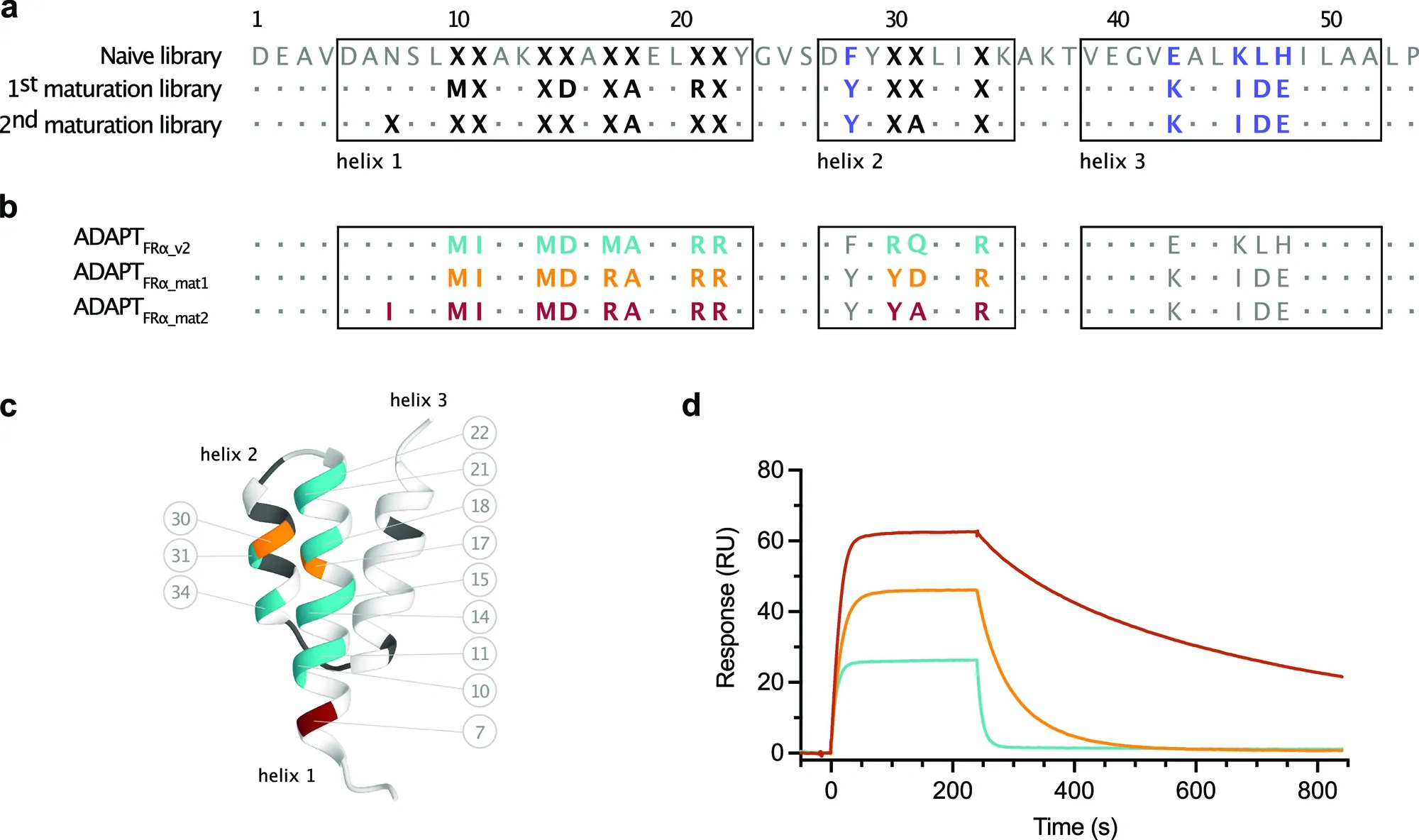
Affinity maturation of an ADAPT against FRα. (a) The design of the naive ADAPT library giving rise to the first-generation FRα-binder, followed by the first and second affinity maturation libraries. Randomized library positions are marked with X (bold), and locked positions are marked with the respective one-letter residue (bold). One residue in helix 2 and four residues in helix 3 (blue) mark scaffold mutations that differ between the HSA-affinity-engineered scaffold ABD035 (naive library) and the stability-engineered scaffold ABD_stable_ (maturation libraries). (b) The respective sequence of the best FRα-binder identified from each library displayed in panel a. (c) Schematic structure of the bispecific ADAPT with the randomized library surface in helices 1 and 2 (turquoise, orange, red) and the HSA-binding surface in helices 2 and 3 (dark gray).^22,23^ Turquoise residues were identified during the naive selections to FRα. Orange and red residues were mutated during the first and second affinity maturation, respectively, leading to a slower dissociation rate from FRα. (d) SPR analysis showing the binding profile of ADAPT_FRα_v2_ (turquoise), ADAPT_FRα_mat1_ (orange), and ADAPT_FRα_mat2_ (red) to immobilized FRα.

**Table 1:** Characterization Summary of the Lead ADAPT Variants throughout the Affinity Maturation^a^

			FRα			HSA		
variant	*T*_m_ (°C)	refolds	*k*_a_ (M^–1^ s^–1^)	*k*_d_ (s^–1^)	*K*_D_ (nM)	*k*_a_ (M^–1^ s^–1^)	*k*_d_ (s^–1^)	*K*_D_ (nM)
ADAPT_FRα_v2_	59	yes	1.7 (±0.4) × 10^6^	1.2 (±0.4) × 10^–1^	79	1.5 × 10^7^	1.0 × 10^–4^	0.0066^a^
ADAPT_FRα_mat1_	64	yes	2.3 (±0.1) × 10^6^	2.3 (±0.2) × 10^–2^	10	6.3 × 10^5^	1.8 × 10^–3^	2.8
ADAPT_FRα_mat2_	47	no	3.9 (±1.6) × 10^6^	2.8 (±0.2) × 10^–3^	0.80	1.6 × 10^6^	1.6 × 10^–3^	1.1
ADAPT22	59	yes	2.6 (±0.2) × 10^6^	2.2 (±0.3) × 10^–3^	0.84	7.7 × 10^5^	1.8 × 10^–3^	2.4

a Thermal stability is described in terms of melting temperature (*T*_m_) and ability to refold after thermal denaturation, as assessed by CD spectroscopy. The equilibrium dissociation constant (*K*_D_) to recombinant FRα and HSA was calculated from the association rate constant (*k*_a_) and the dissociation rate constant (*k*_d_) by fitting six binder concentrations to a 1:1 binding model in SPR analysis (Supporting Information Figure S6). Kinetic parameters to FRα are presented as mean values with standard deviation of three independent experiments.

b Less than 10% dissociation of ADAPT_FRα_v2_ from HSA during the measured time frame in SPR (30 min dissociation), meaning that the real *K*_D_ value is probably lower than the reported value.

### Initial Affinity Maturation to Obtain ADAPT Binders with Improved Off-Rate

After identifying an optimal paratope for simultaneous binding to FRα and HSA, we designed an affinity maturation library based on the amino acid distribution of the sequence cluster containing ADAPT_FRα_v2_ (Supporting Information Figure S3). Four library positions were locked, as they displayed a dominant residue in over 90% of the cluster, while the remaining seven positions were randomized to different degrees (Supporting Information Figure S4). Hydrophobic residues were excluded at specific positions, due to their enrichment in a less thermally stable FRα-binding cluster (Supporting Information Figure S1, green, data not shown). To further promote stability, the scaffold of the library, formerly based on ABD035 (*K*_D,HSA_ = 50–500 fM, *T*_m_ = 58 °C),^45^ was replaced by the stability-engineered ABD_stable_ (*K*_D,HSA_ = 50 nM, *T*_m_ > 80 °C)^33^ via five scaffold mutations, presumably lowering HSA affinity but likely retaining *in vivo* half-life considering the high plasma levels of HSA (600 μM).^20^ The maturation library (Figure 1a) was displayed on phages and selected for improved off-rate to FRα. A first gentle selection round was followed by two stringent rounds at elevated temperature (37 °C), including competitive binding by a large excess of unlabeled FRα to eliminate fast dissociating binders. The selection concluded with a gentle round to recover the enriched binders.

From the deep sequencing of the selection output, 24 enriched ADAPT clones were selected for off-rate screening using biolayer interferometry (BLI). A majority of the clones showed an improved off-rate to FRα, although only to a modest degree (Supporting Information Figure S5). The best clone, ADAPT_FRα_mat1_ (Figure 1b,d), displayed a 5-fold improvement in dissociation rate (*k*_d_ = 2.3 × 10^–2^ s^–1^), resulting in an affinity (*K*_D_) of 10 nM to FRα (Table 1). As expected, the scaffold exchange reduced the binder’s affinity to HSA and increased the melting temperature by 5 °C (Table 1).

To gain more insight into the paratope of ADAPT_FRα_mat1_, an alanine scanning mutagenesis was performed by substituting each library position one-by-one to alanine or to valine for pre-existing alanines. CD spectroscopy showed preserved α-helical structure and high thermal stabilities, thus confirming the structural integrity of all mutans (data not shown). BLI analysis revealed a binding surface predominately located to helix 1 (Supporting Information Figure S7), where substitutions M10A, I11A, M14A, D15A, R17A, and A18V abolished FRα binding, and R21A strongly impaired the interaction. Mutations located in helix 2 or in the final turn of helix 1 (R22A, Y30A, R34A) had a slightly negative effect on the dissociation rate, except for D31A which had no impact on binding. These results align with the deep sequencing data indicating strong residue conservation in helix 1, while helix 2 displayed greater variability (Supporting Information Figures S3 and S4).

### Second Affinity Maturation Utilizing the Leader Sequence to Enlarge the FRα Paratope

Given the critical role of helix 1 in mediating interaction with FRα, we were encouraged to extend the paratope toward the N-terminal by targeting the ADAPT leader sequence (DEAVDANS) in a second affinity maturation library. The leader sequence is not part of the naturally occurring ABD from streptococcal protein G but exists as a remnant from cloning during the initial characterization of this domain.^46^ However, solution NMR data suggest that the three last residues of the leader sequence are involved in the formation of helix 1 (PDB entry 1GJT).^21^ Thus, residue N7, oriented in the same plane as the FRα paratope, was randomized to expand the interaction surface and improve the dissociation kinetics. The remaining library positions were diversified based on the alanine scanning results. Critical helix 1 residues were softly diversified using biased randomization favoring the original amino acid (50–70%) and residues with similar physicochemical properties (Supporting Information Figure S8). In contrast, the library positions in helix 2, as well as the new library position in the leader sequence, were fully randomized using all amino acids except cysteine, proline, methionine, and glycine. Position 31 was locked to alanine due to its lack of contribution to FRα-binding. Phage display selection using this library (Figure 1a) followed the previous strategy but included parallel tracks of different stringency, applying either one or two rounds of off-rate competition using a large excess of unlabeled FRα.

Due to the introduction of a completely new randomized position and its unknown effect on the integrity of the protein, we decided to perform an extended functionality screen of the selection output. Therefore, over 300 ADAPT clones were screened for binding to FRα. Phagemid outputs were pool cloned into a bacterial expression vector and transformed into *E. coli*. Single colonies were cultured in plate format, followed by high-throughput BLI screening of the ADAPT proteins directly from cell supernatant, enabling rapid kinetic assessment of 332 ADAPT clones. Although a majority of the variants displayed functional binding to FRα, only two clones displayed a substantial improvement in dissociation rate, emphasizing the challenges in applying efficient selection pressure in affinity maturation campaigns and the importance of subsequent screening. These two clones were both from the selection track of the greatest stringency, with two subsequent rounds of off-rate competition. Deep sequencing of the selection output revealed that the best clone, ADAPT_FRα_mat2_ (Figure 1b,d), had indeed been enriched over the selection but remained rare in the final round (0.11% out of 925 172 reads). SPR analysis confirmed that ADAPT_FRα_mat2_ exhibited a 100-fold increase in affinity to FRα compared to the first-generation binder (Table 1). Notably, the two affinity-matured binders differed in a single library position, namely, the one in the leader sequence, displaying either an isoleucine (ADAPT_FRα_mat2_, *K*_D_ = 0.80 nM) or a valine (*K*_D_ = 1.6 nM) instead of the parental asparagine in ADAPT_FRα_mat1_ (*K*_D_ = 10 nM). Further kinetic analysis highlighted the combined effect of the first and last library position on the dissociation rate, in which the improved off-rate attributed to isoleucine in the leader sequence is lost if the last library position is mutated from arginine to glutamine (R34Q).

### Stabilization of the Helical Structure to Accommodate for High Affinity without Compromising Biophysical Properties

CD analysis revealed that the affinity-improved binder, ADAPT_FRα_mat2_, had lost its ability to refold after thermal denaturation. Additionally, the binder displayed a reduction in melting temperature by 17 °C (*T*_m_ = 47 °C, Table 1). Thus, the single substitution in the leader sequence (N7I), responsible for the great improvement in FRα affinity, has a notable negative effect on stability. Homology modeling (SWISS-MODEL)^36,37^ of ADAPT_FRα_mat2_ to wild-type ABD (PDB 1GJT) supported the structural role of the leader sequence and suggested that D5 serves as the Ncap by initiating helix 1. It is well-known that the Ncap and the third residue ahead, called N3, play a critical role in α helix stability by forming the N-capping motif. The N-capping motif compensates for the absence of intrahelical hydrogen bonds in the first helical turn, by providing side chains that act as hydrogen bond acceptors.^38,47^ Based on this, we performed rational mutagenesis on ADAPT_FRα_mat2_ by targeting the N-capping motif. The Ncap residue (D5) was retained, with the rationale that aspartate is one of the most frequent Ncap residues in nature. The N3 position (S8) was substituted to Q, N, E, or D (Figure 2a), guided by their high prevalence in naturally occurring α helices when paired with an aspartate Ncap.^38–40^ SPR analysis confirmed that the N3 mutations had no significant impact on the affinity to FRα and HSA. All N3 mutations resulted in improved thermal stabilities, observed in CD spectroscopy by an increase in melting temperature by 8–14 °C (Figure 2b). Furthermore, mutation S8D fully restored the ability to refold after thermal denaturation (Figure 2c). The stability loss induced by N7I can thus be compensated by S8D. The stabilized high-affinity binder containing the S8D mutation was selected for further studies and is hereafter called ADAPT22.

**Figure 2:**
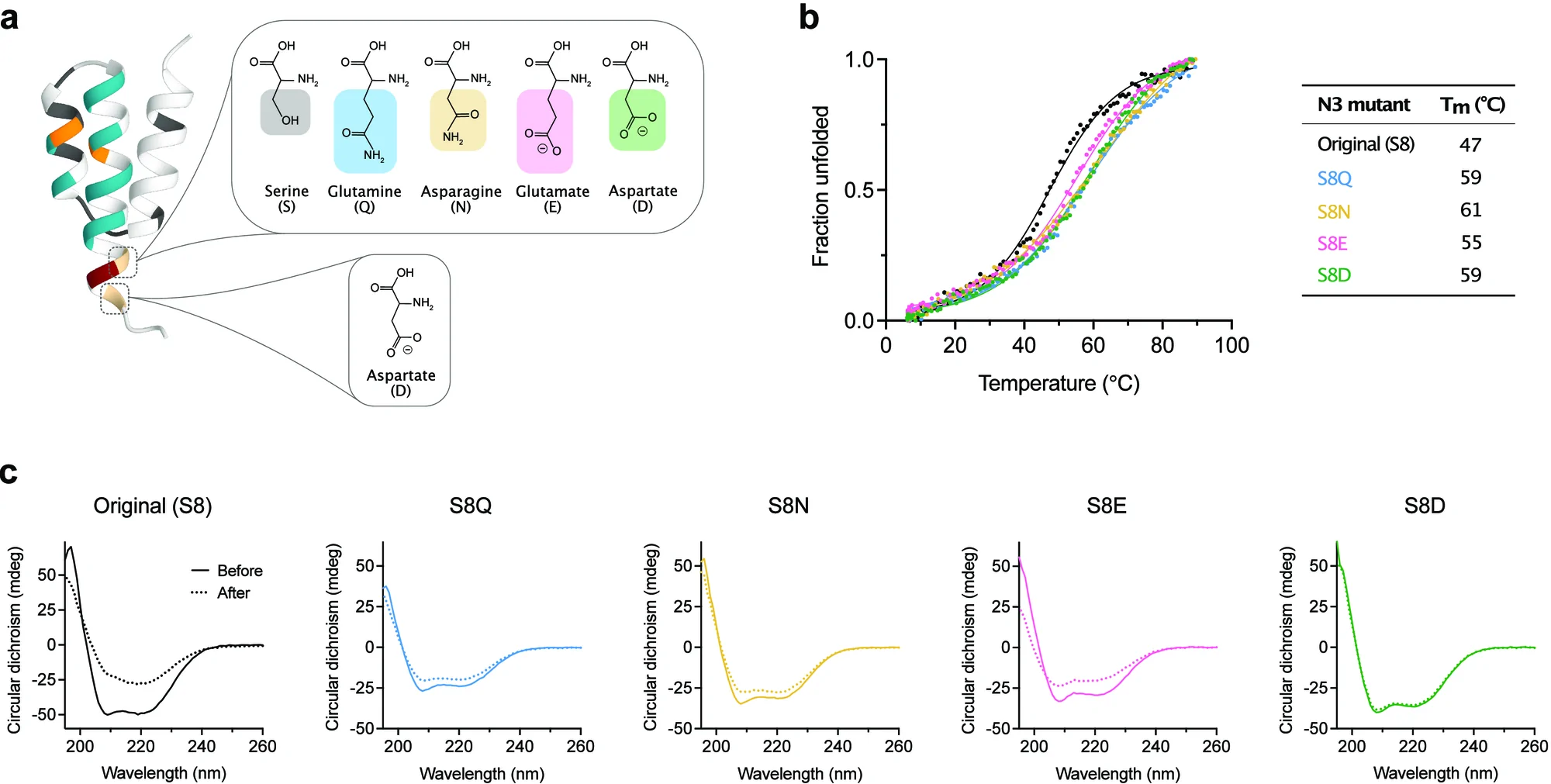
Mutational study aiming to stabilize the N-capping motif in ADAPT_FRα_mat2_. (a) Homology model of ADAPT_FRα_mat2_ (based on template PDB 1GJT). The model suggests that the final four residues of the N-terminal leader sequence (underlined in DEAVDAIS) form the first turn of helix 1 and serves as the N-capping motif. The Ncap (D5) and the mutated N3 position (originally S8, mutated to Q, N, D, or E) are encircled in the model. The chemical structures, with highlighted side chains, are shown for the evaluated amino acids. (b) The melting curves of the different N3 mutants and the corresponding melting temperatures. (c) CD spectra showing the secondary structure of the N3 mutants before (solid line) and after (dotted line) thermal denaturation. The spectra are overlaid to assess refolding ability.

### *In Vitro* Characterization of ADAPT22

Following the affinity maturation and structure stabilization, the final binder was thoroughly characterized. ADAPT22 demonstrated high affinity in SPR analysis, with equilibrium dissociation constants (*K*_D_) of 0.84 nM to human FRα and 2.4 nM to HSA (Table 1). The binder displayed cross-species reactivity to murine targets but with lower affinities (*K*_D_ = 77 nM to mouse FRα, *K*_D_ = 9.9 nM to mouse serum albumin (MSA); Supporting Information Figure S9). High specificity for FRα was maintained throughout the maturation, with minimal cross-reactivity to all other human folate receptor isoforms (Figure 3a). Epitope binning experiments identified an epitope distinct from the folate ligand binding site (Figure 3b) but partially overlapping with mirvetuximab (Figure 3c), a clinically approved anti-FRα antibody with an apparent affinity of ≤ 0.1 nM.^48^ While ADAPT22 could not bind FRα preoccupied by mirvetuximab, the antibody was able to displace the much smaller ADAPT binder, indicating partially shared but nonidentical epitopes. This interpretation is further reinforced by the observation that ADAPT22, but not mirvetuximab, is able to bind an epitope on murine FRα (Supporting Information Figure S10).

**Figure 3:**
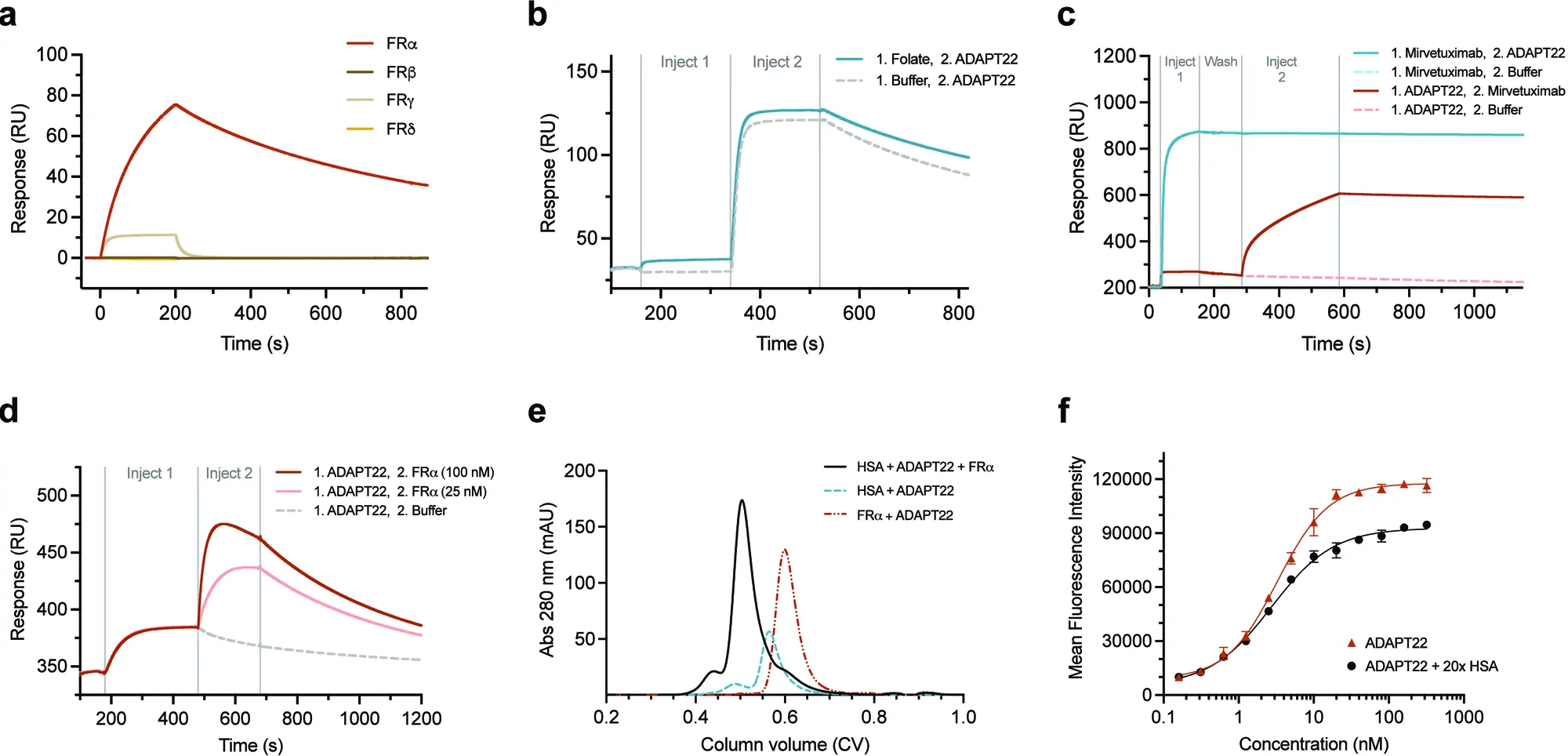
*In vitro* characterization of ADAPT22. (a) Cross-reactivity to all human isoforms of the folate receptor, showing binding with high affinity only to the cancer-associated α isoform. ADAPT22, conjugated to a C-terminal biotin, is captured on a streptavidin-coated surface, and the folate receptors are injected as analytes (100 nM). (b) Binding of ADAPT22 (50 nM) to immobilized FRα (617 RU) in the absence (dotted gray) or presence (solid blue) of the natural ligand folate (2000 nM). (c) Epitope binning of ADAPT22 (500 nM) and mirvetuximab (500 nM) to immobilized FRα (529 RU), in which the ADAPT is injected first followed by a second injection of the antibody or vice versa. The blue lines are overlapping. (d–f) Simultaneous bispecificity of ADAPT22 to HSA and FRα. (d) SPR was used to capture ADAPT22 on immobilized HSA (852 RU) followed by an injection with 25 nM or 100 nM FRα. (e) Analytical SEC was performed on ADAPT22 in complex with HSA (dashed blue), FRα (dashed red), or both HSA and FRα (solid black) in equimolar ratios. (f) Flow cytometry of FRα-expressing SKOV-3 cells stained with ADAPT22 alone or ADAPT22 preincubated with 20-fold molar excess of HSA, performed in triplicates and reported as mean fluorescence intensity with standard deviations. The data were fitted to a sigmoidal function, from which the on-cell affinity was calculated to 3.3 nM in the absence of HSA and to 2.7 nM in the presence of HSA.

Simultaneous binding to FRα and HSA is of outmost importance, ensuring that circulating, albumin-associated ADAPT can effectively target FRα at the tumor site. Hence, the simultaneous nature of ADAPT22 was characterized by three different methods. First, simultaneous bispecificity was verified by SPR, in which ADAPT22 was captured on immobilized HSA, followed by a second binding event upon injection with FRα (Figure 3d). Analytical SEC further demonstrated simultaneous binding, as an equimolar mixture of ADAPT22, FRα, and HSA eluted as a single peak corresponding to the ternary complex (Figure 3e). This shows that ADAPT22 forms a stable complex with FRα and HSA, without steric hindrance or target bias. Thereafter, binding to endogenously expressed FRα on human cancer cell lines was assessed by flow cytometry. Initial experiments confirmed specific binding of ADAPT22 to FRα-positive ovarian- and breast-derived cancer cell lines (IGROV-1, SKOV-3, MCF-7) with no binding to negative controls (BxPC-3, Ramos, Jurkat) (Supporting Information Figures S11 and S12). Finally, biotinylated ADAPT22 in varying concentrations, either alone or preincubated with 20-fold excess of HSA, was added to FRα-expressing SKOV-3 cells and the binding was monitored by fluorophore-labeled streptavidin. The on-cell affinity was determined to 3.3 nM and 2.7 nM in the absence and presence of HSA, respectively, indicating that albumin association does not impair FRα binding (Figure 3f). Despite the highly similar on-cell affinities in the absence and presence of HSA, lower fluorescent binding signals were observed for ADAPT22 preincubated with HSA. This observation may be due to steric hindrance during the indirect labeling with fluorescent streptavidin, where bound HSA potentially limits access to the C-terminal biotin of ADAPT22.

Taken together, ADAPT22 demonstrates an exceptional ability to simultaneously interact with FRα and HSA, a notable achievement for an affinity protein of such small size and an essential feature for its therapeutic potential.

### Radiolabeling and *in Vitr*o Studies

For quantitative assessment of binding, internalization, and biodistribution, ADAPT22 was site-specifically radiolabeled with technetium-99m tricarbonyl. Radiolabeling was efficient and consistently yielded a radiochemical purity exceeding 96% (Supporting Information Table S3). No significant release of radioactivity was observed, confirming the stability of the radiolabel.

To verify that radiolabeling did not alter target binding, the binding specificity of [^99m^Tc]­Tc­(CO)_3_-ADAPT22 was evaluated using FRα-positive IGROV-1 and SKOV-3 cells, with PC-3 cells serving as an FRα-negative control.^49^ Preincubation of IGROV-1 and SKOV-3 cells with an excess of unlabeled ADAPT22 markedly reduced cell-associated activity relative to cells incubated with [^99m^Tc]­Tc­(CO)_3_-ADAPT22 alone, showing saturable and FRα-dependent binding (Supporting Information Figure S13). Uptake normalized to cell number was higher in IGROV-1 than in SKOV3-cells, consistent with earlier reported values of FRα-expression in ovarian carcinoma cell lines.^48,50^ The low uptake of [^99m^Tc]­Tc­(CO)_3_-ADAPT22 in FRα-negative PC-3 cells was not specific.

Internalization studies in IGROV-1 cells (Supporting Information Figure S14) showed that [^99m^Tc]­Tc­(CO)_3_-ADAPT22 is internalized, an essential feature for therapeutic efficacy of intracellularly acting payloads. Notably, ADAPT22 retained its capacity in the presence of HSA, supporting its functionality under physiologically relevant conditions.

Based on the binding and internalization results, IGROV-1 cells were selected as the FRα high-expressing/positive model and PC-3 cells as the low-expressing/negative model for *in vivo* studies.

### *In Vivo* Studies

To determine whether engineering dual affinity toward serum albumin and FRα can modulate *in vivo* pharmacokinetics, we compared the biodistribution properties of the novel construct ADAPT22 with the clinically validated HER2-binding ADAPT6 in NMRI mice, with [^99m^Tc]­Tc­(CO)_3_-ADAPT6 serving as a reference of a non-albumin binding ADAPT. ADAPT22 showed a markedly extended residence time in blood compared with [^99m^Tc]­Tc­(CO)_3_-ADAPT6 (Figure 4a). This prolonged systemic circulation is consistent with the albumin-binding functionality engineered into ADAPT22. At 4 h postinjection, ADAPT22 demonstrated a significantly (*p* < 0.001, unpaired *t* test) higher uptake in all studied organs and tissues relative to ADAPT6, except kidneys, where it was 24-fold lower (Figure 4b). These data indicate that the engineered albumin affinity effectively decreases renal uptake while maintaining prolonged circulating levels due to albumin binding.

**Figure 4:**
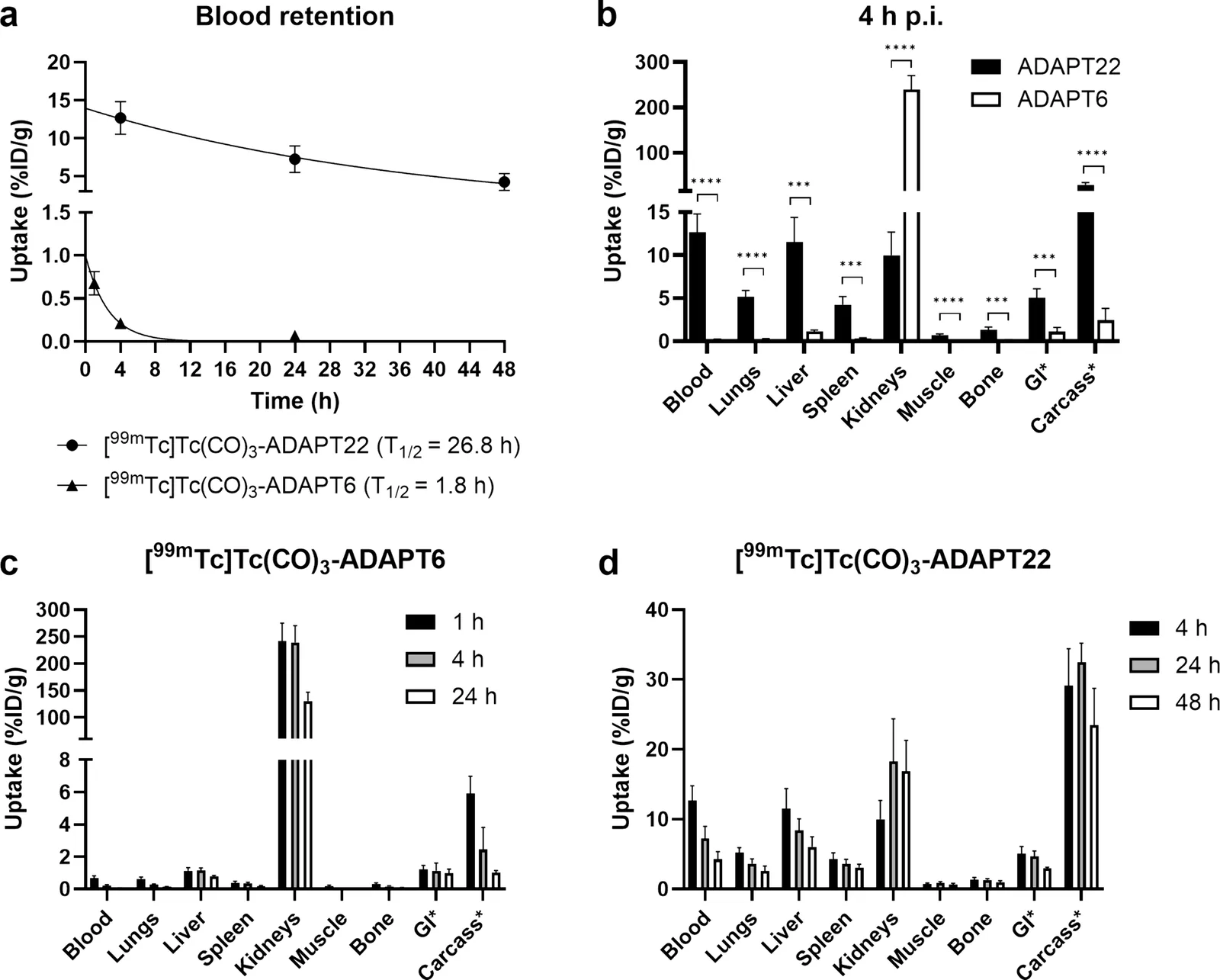
Comparison of blood retention and biodistribution of [^99m^Tc]­Tc­(CO)_3_-labeled ADAPT22 and ADAPT6 in normal NMRI mice. (a) Blood retention and (b) biodistribution at 4 h postinjection. Biodistributions of [^99m^Tc]­Tc­(CO)_3_-ADAPT6 at 1, 4, and 24 h p.i. and of [^99m^Tc]­Tc­(CO)_3_-ADAPT22 at 4, 24, and 48 h p.i. are shown in (c) and (d), respectively. Blood retention was estimated using a one-phase decay model (plateau constrained to zero). Uptake is presented as %ID/g ± SD except for the gastrointestinal tract and carcass, which are shown as %ID/sample ± SD (*n* = 4). Statistical significance is indicated by asterisks (***p < 0.001; ****p < 0.0001, unpaired *t* test).

To investigate the tumor-targeting properties of ADAPT22, we studied its biodistribution in Balb/c nu/nu mice bearing FRα-expressing IGROV-1 xenografts (Figure 5). Preinjection of a large excess of unlabeled ADAPT22 resulted in a significant reduction in tumor accumulation of [^99m^Tc]­Tc­(CO)_3_-ADAPT22 (*p* < 0.0001, one-way ANOVA; Figure 5a,b), demonstrating that the tumor uptake is saturable. Consistent with receptor-mediated targeting, uptake in IGROV-1 xenografts was substantially higher than in FRα-negative PC-3 xenografts, showing dependence on FRα expression. Both control groups, PC-3 tumors and IGROV-1 tumors under blocking conditions, showed similarly low tumor uptake, confirming complete receptor saturation in the blocking group and supporting the specificity of ADAPT22 for FRα.

**Figure 5:**
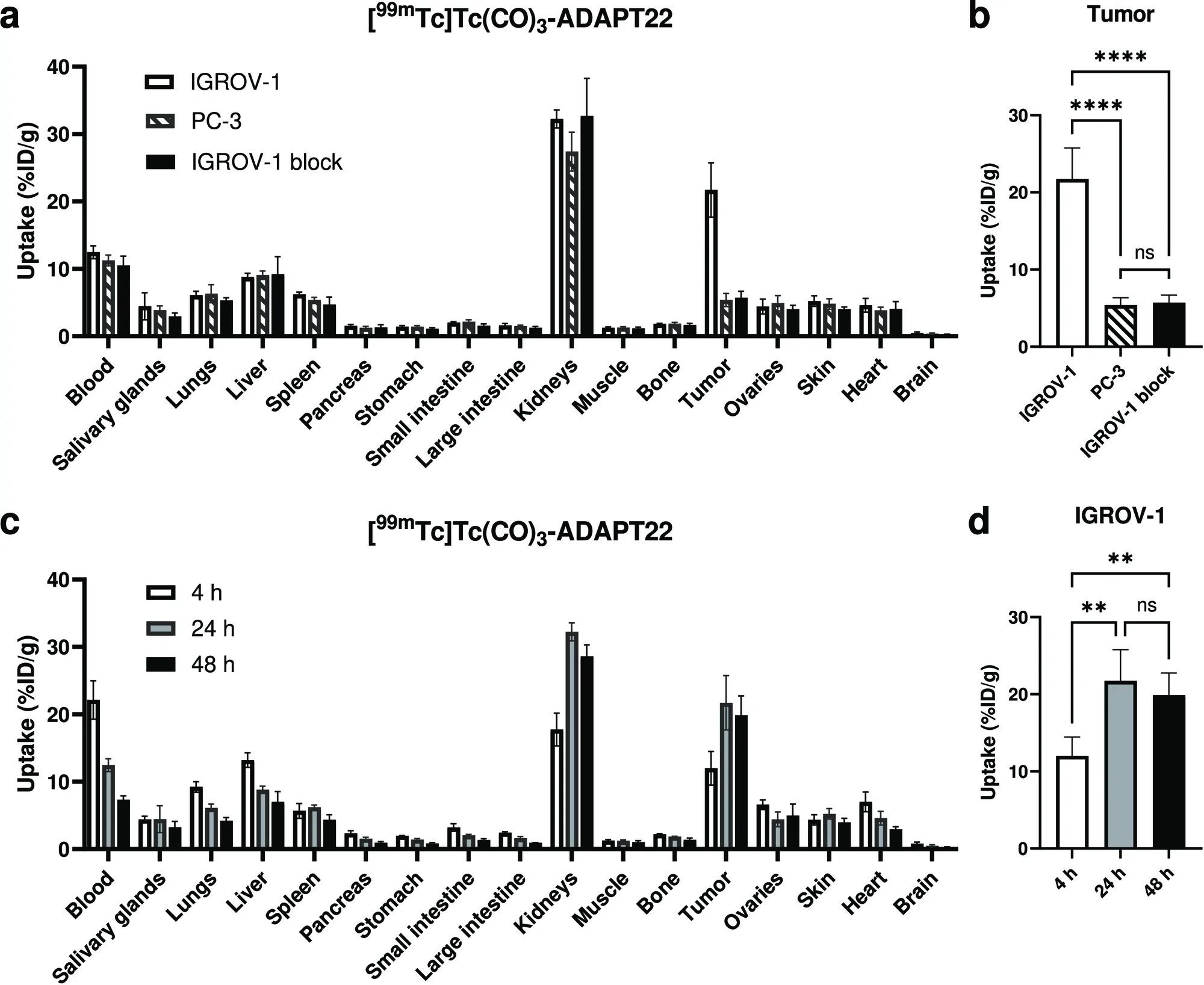
Biodistribution of [^99m^Tc]­Tc­(CO)_3_-ADAPT22 in BALB/c nu/nu mice bearing tumor xenografts. (a) Biodistribution at 24 h postinjection (p.i.) in mice bearing FRα-positive IGROV-1 xenografts, FRα-negative PC-3 xenografts, or in a blocking group preinjected with excess unlabeled ADAPT22 18 h prior to tracer administration (IGROV-1 block). (b) Comparison of tumor-targeting specificity at 24 h p.i. (c) Biodistribution at 4, 24, and 48 h p.i. in mice bearing IGROV-1 xenografts. (d) Comparison of tumor uptake in IGROV-1 xenografts over time. Uptake is presented as %ID/g ± SD (*n* = 4–5). Statistical significance is indicated by asterisks (**p < 0.01; ****p < 0.0001; ns, not significant; one-way ANOVA).

Biodistribution measured at 4, 24, and 48 h p.i. further showed an extended blood retention in mice bearing IGROV-1 xenografts (Figure 5c), consistent with the data in NMRI mice. Uptake in highly perfused organs such as liver and lungs decreased over time following a decline in blood activity. Renal uptake was 18 ± 3 %ID/g at 4 h, peaked at 32 ± 1 %ID/g at 24 h, and remained close to this level at 48 h. Tumor uptake followed a similar pattern, with 12 ± 3 %ID/g at 4 h and a maximum of 22 ± 4 %ID/g at 24 h, followed by sustained retention of 20 ± 3 %ID/g at 48 h (Figure 5d). This stability over time indicates durable tumor retention despite systemic clearance. From 24 h onward, tumor uptake exceeded that of all other organs and tissues except the kidneys.

To provide a normalized assessment of tissue distribution over time, tissue-to-blood ratios were calculated (Supporting Information Tables S4 and S5). The tissue-to-blood ratios reflected the different distribution profiles of the two constructs. For ADAPT22, the ratios were lower, consistent with its longer residence in the circulation. In contrast, ADAPT6 showed substantially higher tissue-to-blood ratios in most organs at early time points due to its rapid blood clearance.

Micro-SPECT/CT imaging of FRα expression using [^99m^Tc]­Tc­(CO)_3_-ADAPT22 in tumor-bearing mice at 24 h p.i. confirmed the biodistribution data. Imaging showed extended blood circulation and clear visualization of IGROV-1 xenografts (Figure 6). The tumor uptake was visibly reduced when the mouse was preinjected with a large excess of unlabeled ADAPT22 (Figure 6a) or when compared with FRα-negative PC-3 xenograft (Figure 6b).

**Figure 6:**
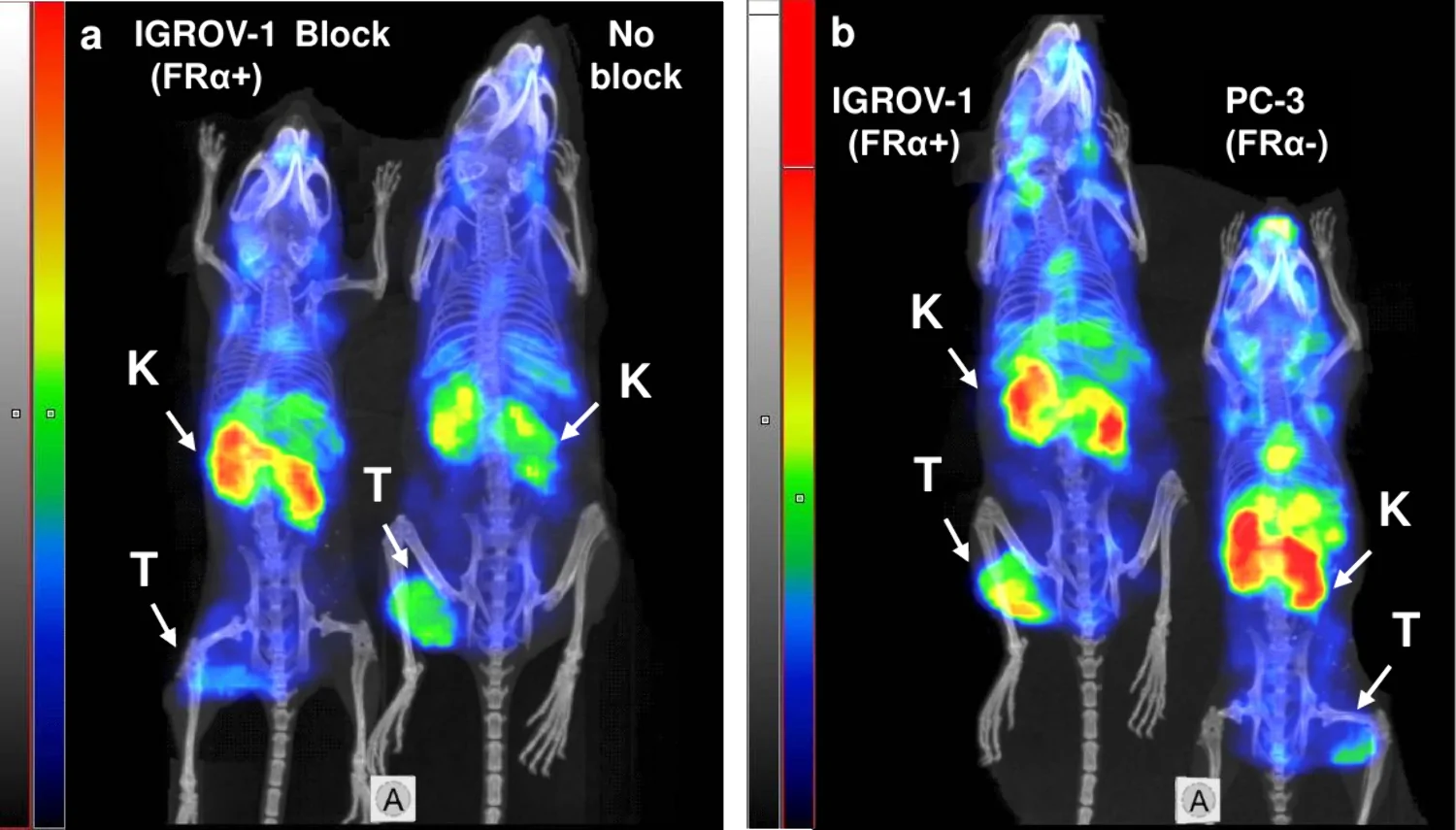
Micro-SPECT/CT imaging of FRα expression in BALB/c nu/nu mice using radiolabeled [^99m^Tc]­Tc­(CO)_3_-ADAPT22 (injected protein dose 2 μg, comparable injected activities) at 24 h p.i. (a) Both mice bearing FRα-positive IGROV-1 xenografts, where a control mouse (left) was preinjected with a large excess of unlabeled ADAPT22 18 h before the injection of the tracer. (b) Mouse bearing FRα-positive IGROV-1 xenograft (left) and mouse bearing FRα-negative PC-3 xenograft (right). Arrows are pointing at kidneys (K) and tumors (T).

Together, these data show that ADAPT22 achieves the intended biodistribution and tumor-targeting properties, characterized by prolonged circulation and significantly reduced kidney uptake relative to the non-albumin binding control (ADAPT6), together with FRα-specific, saturable, and sustained tumor uptake.

## Discussion

FRα is an attractive target in ovarian carcinoma, given its frequent and high expression in advanced stage tumors and limited expression in healthy tissues. Herein, we describe the development of a dual-surfaced ADAPT with simultaneous binding to FRα and HSA. The ADAPT is a robust scaffold protein derived from the ABD, comprising of only 54 amino acids (6 kDa). We have kept the scaffold’s native binding to albumin and introduced a novel binding surface to FRα on the opposite side of the scaffold (Figure 1c). The aim of the dual binding surfaces was to integrate tumor targeting and half-life extension within a single-domain protein, thus keeping the size to a minimum.

A dual targeting FRα- and HSA-binding ADAPT was identified by phage display selections using a bispecific library (Figure 1). Deep sequencing of the selection output identified two FRα-binding clusters, but only one supported simultaneously binding to both targets. This likely reflects spatial constraints imposed by the small size of the ADAPT scaffold (6 kDa) relative to HSA (67 kDa) and FRα (27 kDa), with the two binding sites separated by only ∼14 Å (measured in ChimeraX, PDB 1GJT). The high-nanomolar affinity of the lead candidate (ADAPT_FRα_v2_, *K*_D_ = 79 nM) suggested that further affinity maturation would be required for therapeutic applications. Although the first affinity maturation yielded improved binders, the best variant (ADAPT_FRα_mat1_) only reached a moderate affinity of 10 nM. A subsequent alanine scanning localized the paratope primarily to helix 1, where substitutions in the first seven library positions severely reduced binding. We hypothesized that the affinity could be improved by expanding the relatively small binding interface. Given its proximity to the paratope, the N-terminal leader sequence was exploited for this purpose in a second round of affinity maturation. Notably, a single substitution (N7I) in the leader sequence ultimately resulted in a 100-fold improvement in affinity to FRα (ADAPT_FRα_mat2_, *K*_D_ = 0.80 nM). This underscores the functional importance of the leader sequence and motivates its use in future ADAPT library designs.

The ADAPT scaffold is inherently stable and capable of refolding after thermal denaturation,^33,45^ thereby tolerating harsh conditions during radiolabeling and peptide synthesis. However, this characteristic was lost for the affinity improved variant ADAPT_FRα_mat2_. The reduced stability, caused by the N7I substitution, may have compromised clone recovery during selection, possibly explaining the low enrichment (0.11% of total reads). To counteract the instability introduced by N7I, we sought to stabilize the adjacent N-capping motif, formed by the terminal residues of the leader sequence. The best performing substitution S8D increased the melting temperature by 12 °C and restored the refolding capacity (Figure 2), indicating that a short negatively charged side chain is the optimal hydrogen bond acceptor in the N-capping motif of helix 1. These findings highlight that the leader sequence, despite being a cloning remnant,^46^ has an important structural role in the ADAPT scaffold, and demonstrates that it can be engineered to enhance stability. This is consistent with previous findings showing that truncation of the leader sequence impairs expression, likely attributed to stability issues.^51^

Importantly, the scaffold stabilization did not significantly affect the binding to FRα or HSA. The final candidate, ADAPT22, demonstrated subnanomolar affinity to FRα paired with a slightly lower affinity to HSA (Table 1). Binding to FRα was not blocked by folate (Figure 3b), reducing the risk of competitive interference with the natural ligand. SPR analysis further indicated that ADAPT22 recognizes a unique epitope on FRα that is not conserved across folate receptor isoforms, despite sequence homology of up to 80%.^44^ Importantly, no binding was detected to FRβ or FRδ, while a very weak interaction with FRγ was observed (Figure 3a). This interaction is likely not biologically relevant, given its low affinity and the fact that FRγ is a secreted, nonmembrane-anchored isoform.^52^ The high specificity of ADAPT22 supports therapeutic development by minimizing off-tumor binding and thereby reducing exposure to healthy tissues.

Due to the high abundance of albumin in blood, the ADAPT will likely be HSA-associated when it reaches the tumor, making simultaneous binding to HSA and FRα essential. This was successfully demonstrated *in vitro* by the ability of ADAPT22 to form a ternary complex with both target proteins in SPR (Figure 3d) and SEC analysis (Figure 3e). Further, flow cytometry confirmed binding of HSA-saturated ADAPT22 to cell surface-expressed FRα without any negative impact on affinity (Figure 3f).

Finally, the capacity of ADAPT22 to bind albumin and FRα simultaneously was demonstrated *in vivo* in a mouse model. In NMRI mice, [^99m^Tc]­Tc­(CO)_3_-ADAPT22 exhibited a substantially longer blood retention than the non-albumin-binding control, [^99m^Tc]­Tc­(CO)_3_-ADAPT6, demonstrating effective association to MSA (Figure 4). The long residence of [^99m^Tc]­Tc­(CO)_3_-ADAPT22 was also confirmed in another mouse strain, BALB/C nu/nu. Specific tumor targeting was confirmed by accumulation in FRα-positive xenografts, compared to FRα-negative controls (Figure 5). Given the high serum concentration of MSA (∼450 μM),^53^ it may be presumed that most ADAPT22 is associated with MSA upon tumor interaction, although this was not directly assessed. This study represents the first *in vivo* evaluation of the bispecific ADAPT platform. It demonstrates that dual binding surfaces can be successfully integrated into a single-domain ADAPT, while maintaining their individual and simultaneous functionality *in vivo*.

In previous studies of ADAPT6, a comparison of the technetium-99m tricarbonyl label with the residualizing label indium-111 and the nonresidualizing label iodine-125 (Supporting Information Table S6) suggests that technetium-99m tricarbonyl shows residualizing properties comparable to indium-111 up to 4 p.i. However, the lower kidney retention observed at 24 h for the technetium-99m tricarbonyl label suggests that some label may be released from the cells over time, which should be considered when interpreting tissue distribution data at late time points.

The marked reduction in renal accumulation indicates that dual affinity engineering successfully mitigates the high kidney reabsorption typically associated with small protein-based targeting agents. Accordingly, ADAPT22 has an apparent potential for targeted delivery of cytotoxic drugs. Notably, an ABD-fused HER2-targeting affibody Z_HER2:2891_-ABD-E_3_-mcDM1, conjugated to the maytansine derivative DM1, resulted in efficient treatment of human ovarian cancer xenografts in mice without detectable kidney or liver toxicity.^54^ Compared to this construct, ADAPT22 exhibit lower renal uptake (32 ± 1 vs 56 ± 9 %ID/g, 24 h after injection) and higher tumor uptake (22 ± 3 vs 6.9 ± 0.3 %ID/g), suggesting that an ADAPT22-DM1 conjugate could improve therapeutic efficacy.

Although ADAPT22 cross-reacts with murine FRα (*K*_D_ = 77 nM), the affinity is considerably lower than for human FRα. Future assessment of on-target toxicity to healthy tissues would therefore require transgenic mouse models to be of relevance. The distribution of FRα in normal tissues is broadly similar in mice and humans, with high kidney expression in both species and similar expression in choroid plexus.^5,55,56^ Similarly, folate-specific uptake has also been demonstrated in the salivary glands of mice.^57^ However, species-specific differences have been reported, with lower expression levels in mouse heart, lung, liver and intestine compared with humans.^5^ Therefore, while mouse models provide useful information regarding the biodistribution of FRα-targeting tracers, they do not fully predict normal tissue uptake in humans.

It should also be noted that receptor accessibility from the circulation may differ from total tissue expression. FRα is normally expressed on the apical side of healthy polarized epithelia and is therefore largely inaccessible to the bloodstream.^52,58^ However, in tumors, FRα becomes accessible to circulating targeting agents due to loss of polarity and disruption of intercellular junctions.^55^ Nevertheless, the expression of FRα in the proximal tubule of the kidneys is relevant to consider due to the renal reabsorption of ADAPT22. It should be noted, however, that renal reabsorption is a common feature for small proteins, mediated by promiscuous scavenger receptors in the proximal tubule,^59^ and should not solely be attributed to FRα-mediated reabsorption. Future studies should explore the tunability of the albumin binding, and whether decreasing the albumin dissociation rate may prevent renal filtration and further reduce renal accumulation.

Immunogenicity remains an important consideration for therapeutic development and will require thorough investigation. Encouragingly, clinical and preclinical studies of ADAPT-based molecules, such as ADAPT6, have demonstrated favorable safety and tolerability profiles, with no toxicity or other adverse effects detected.^31^ Similarly, the therapeutic izokibep, carrying an albumin-binding domain, has shown good tolerability in clinical studies, at doses up to 160 mg every 2 weeks for 24 weeks, supporting the potential of the scaffold for repeated dosing in humans.^60^

The rationale for integrating dual binding surfaces into a single-domain ADAPT was to maintain a minimal scaffold size, enabling straightforward synthesis and optimizing tumor penetration. The ADAPT is only 6 kDa, but previous studies suggest that size can influence tumor uptake, and distribution within the tumor, even at this size scale.^61–64^ It should be noted that albumin association increases the apparent size, which likely influences tumor penetration. However, the reversible nature of the affinity-based binding could be beneficial as it may allow for partial dissociation within the tumor. This could potentially facilitate the diffusion of free ADAPT, an aspect that should be further investigated using tumor organoid models. Supporting this, albumin binding did not impair spheroid penetration of Humabodies^65^ or tumor penetration of an albumin-binding Fab *in vivo*,^66^ suggesting that albumin binding is a viable strategy for small therapeutic formats.

## Conclusions

In summary, we have developed a single-domain ADAPT with bispecificity for FRα and HSA, thereby integrating tumor targeting and half-life extension within one of the smallest scaffold proteins reported to date. The presented results on ADAPT22 demonstrate a high scaffold stability, simultaneous binding to FRα and HSA, highly FRα-specific tumor targeting, and prolonged blood retention *in vivo*. Taken together, these results provide a strong foundation for advancing ADAPT22 as a therapeutic candidate. Future studies should focus on payload conjugation and comprehensive preclinical evaluation of efficacy and safety.

## Supplementary Material



## Notes

Additional experimental details, deep sequencing data, initial characterization of the first-generation binder, SPR data of the evaluated protein variants, BLI data of the alanine scanning mutagenesis, flow cytometry data of ADAPT22 to human cancer cell lines, radiochemical yield and label stability, cell-associated activity and internalization of [^99m^Tc]­Tc­(CO)_3_-ADAPT22, tissue-to-blood ratios, comparison of kidney uptake of ADAPT6, blood retention plotted on a semilogarithmic scale (PDF)

## Abbreviations

ABD: albumin-binding domain
ADAPT: ABD-derived affinity protein
FRα: folate receptor alpha
HSA: human serum albumin
MSA: mouse serum albumin
