# Supplementary material for: Integrating Tumor Targeting and Half-Life Extension into a Dual-Surfaced Single-Domain ADAPT Protein
Source: Mol Pharm. 2026 Jul 28;23(9):4743–57. doi: 10.1021/acs.molpharmaceut.6c00683 (PMC13556137; doi:10.1021/acs.molpharmaceut.6c00683)
Supplement: Supplementary file 1 [file mp6c00683_si_001.pdf]

Supporting Information

Integrating tumor targeting and half-life extension into a dual-surfaced single-domain ADAPT protein

Emma Larsson[§,a], Moeen Ud-din[§,b], Athanasios Bitzios[b], Enrique Recasens Perez-Vera[b], Hanna Tegel[a], Anna Orlova[c], Vladimir Tolmachev[b], Anzhelika Vorobyeva[b], Sophia Hober[a]

[a]Department of Protein Science, SciLifeLab, KTH Royal Institute of Technology, Stockholm 106 91, Sweden [b]Department of Immunology, Genetics and Pathology, Uppsala University, Uppsala 751 85, Sweden

[c]Department of Medicinal Chemistry, Uppsala University, Uppsala 751 23, Sweden

[§]These authors contributed equally to this study.

Supplementary Table S1. Information about the target protein used during the selections. The construct sequence includes the full-length extracellular domain of human FRα, only omitting the signal peptide and the propeptide in the construct design. An HPC4-tag was added to the C-terminus to allow for purification.

Protein name	Gene name	Uniprot ID	Construct sequence	Sequence length (aa)	Molecular weight (Da)
FRα	FOLR1	P15328	RIAWARTELLNVCMNAKHHKE<br>KPGPEDKLHEQCRPWRKNACC<br>STNTSQEAHKDVSYLYRFNWN<br>HCGEMAPACKRHFIQDTCLYE<br>CSPNLGPWIQQVDQSWRKERV<br>LNVPLCKEDCEQWWEDCRTSY<br>TCKSNWHKGWNWTSGFNKCA<br>VGAACQPFHFYFPTPTVLCNEI<br>WTHSYKVSNYSRGSGRCIQMW<br>FDPAQGNPNEEVARFYAAAME<br>NLYFQGEDQVDPRLIDGK	228	26787

Supplementary Table S2. Strategies for phage display selection against the target FRα using **a)** the naive ADAPT library, **b)** the first affinity maturation library, and **c)** the second affinity maturation library. The concentration of biotinylated FRα (with target incubation time in parenthesis), the selection temperature, and the number of washes is displayed for each selection round. The selections were carried out in an automatic magnetic bead handling device, with each wash conducted for 30 s at 1500 rpm, unless stated otherwise. Extended off-rate washes were applied in the affinity maturations, by the addition of 250 – 500x molar excess of non-biotinylated FRα in the third to last wash to prevent rebinding of fast dissociating ADAPTs.

a

	Phage input	Target concentration (incubation time)	Temperature	No. of washes	Track
**Round 1**	Naive library	150 nM (2 h)	RT	2x	R1_T1
**Round 2**	R1_T1	100 nM (1.5 h)	RT	4x	R2_T1
**Round 3**	R2_T1	50 nM (1 h)	RT	6x	R3_T1
**Round 4**	R3_T1	25 nM (1 h)	RT	12x	R4_T1

b

	Phage input	Target concentration (incubation time)	Temperature	No. of washes	Off-rate wash	Track
**Round 1**	Maturation library 1	100 nM (2 h)	RT	4x		R1_T1
**Round 2**	R1_T1	25 nM (1.5 h)	37 °C	15x		R2_T1
**Round 3**	R2_T1	5 nM (1 h)	37 °C	10x	1.25 µM unlabeled FRα 2 h, 37°C	R3_T1
	R2_T1				1.25 µM unlabeled FRα 18 h, RT	R3_T2
**Round 4**	R3_T1	50 nM (1.5 h)	RT	9x		R4_T1
	R3_T2					R4_T2

c

	Phage input	Target concentration (incubation time)	Temperature	No. of washes	Off-rate wash	Track
**Round 1**	Maturation library 2	50 nM (2 h)	37 °C	5x		R1_T1
**Round 2**	R1_T1	5 nM (1.5 h)	37 °C	10x	2.5 µM unlabeled FRα 20 h, RT	R2_T1
	R1_T1				2.5 µM unlabeled FRα 4 h, 37°C	R2_T2
**Round 3**	R2_T1	5 nM (1 h)	37 °C	10x (1500 rpm) + 5x (2000 rpm)		R3_T1
	R2_T2					R3_T2
	R2_T1	1 nM (1 h)	37 °C	15x	0.5 µM unlabeled FRα 3 h, 37°C + 17 h, RT	R3_T3
	R2_T2				0.5 µM unlabeled FRα 20 h, RT	R3_T4

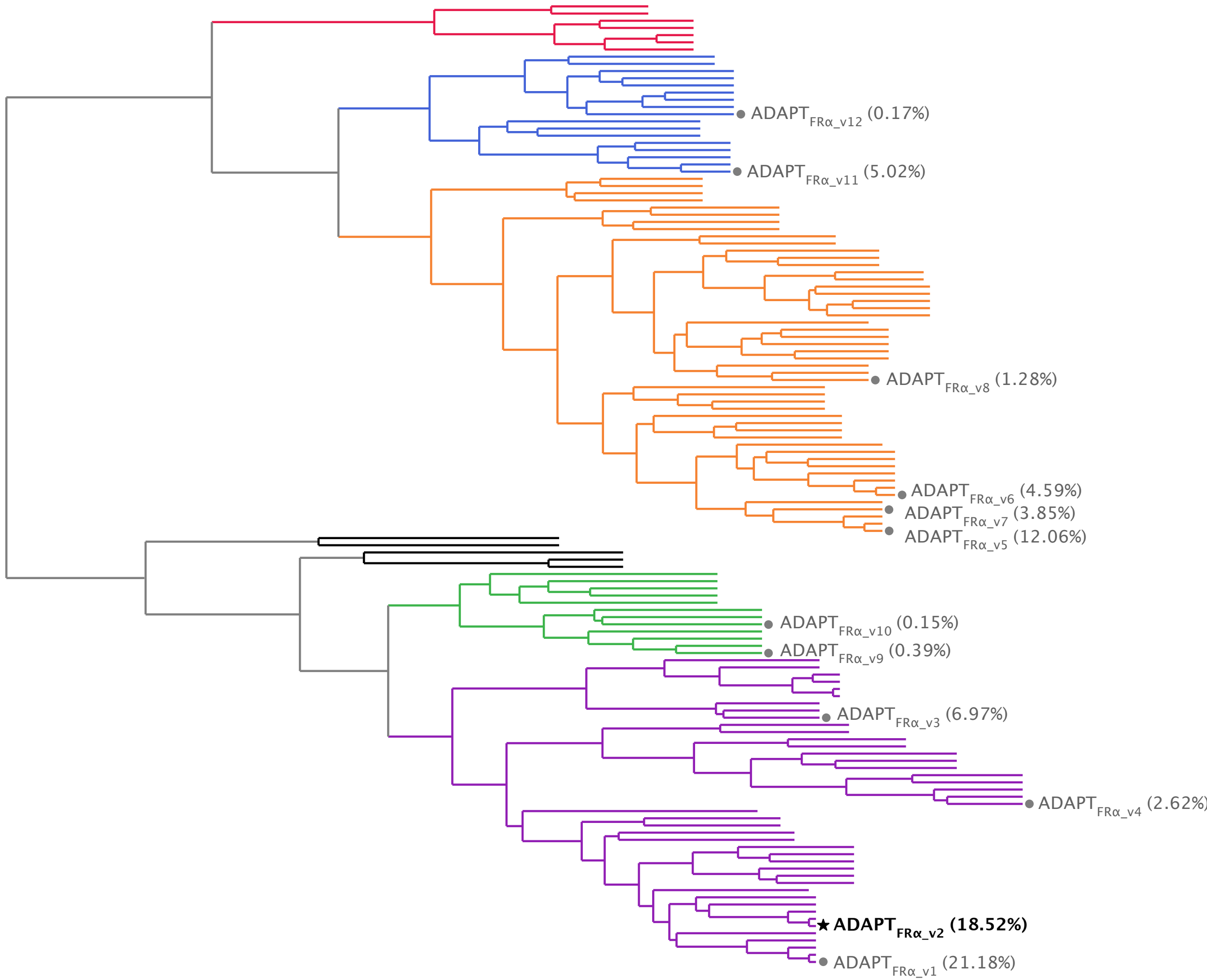

Supplementary Figure S1. Dendrogram of the naive selection output against FRα based on deep sequencing data. The 134 most abundant ADAPT sequences (≥10 counts) from the final selection round have been clustered based on sequence similarity. The ADAPT variants selected for initial characterization are marked together with their occurrence in the deep sequencing data of the final round (out of 102661 total reads). Binders to FRα was found in the green and purple cluster, but only ADAPTs from the purple cluster could interact with FRα and HSA simultaneously. The best performing variant, ADAPT$_{FR\alpha_v2}$, is marked with a star.

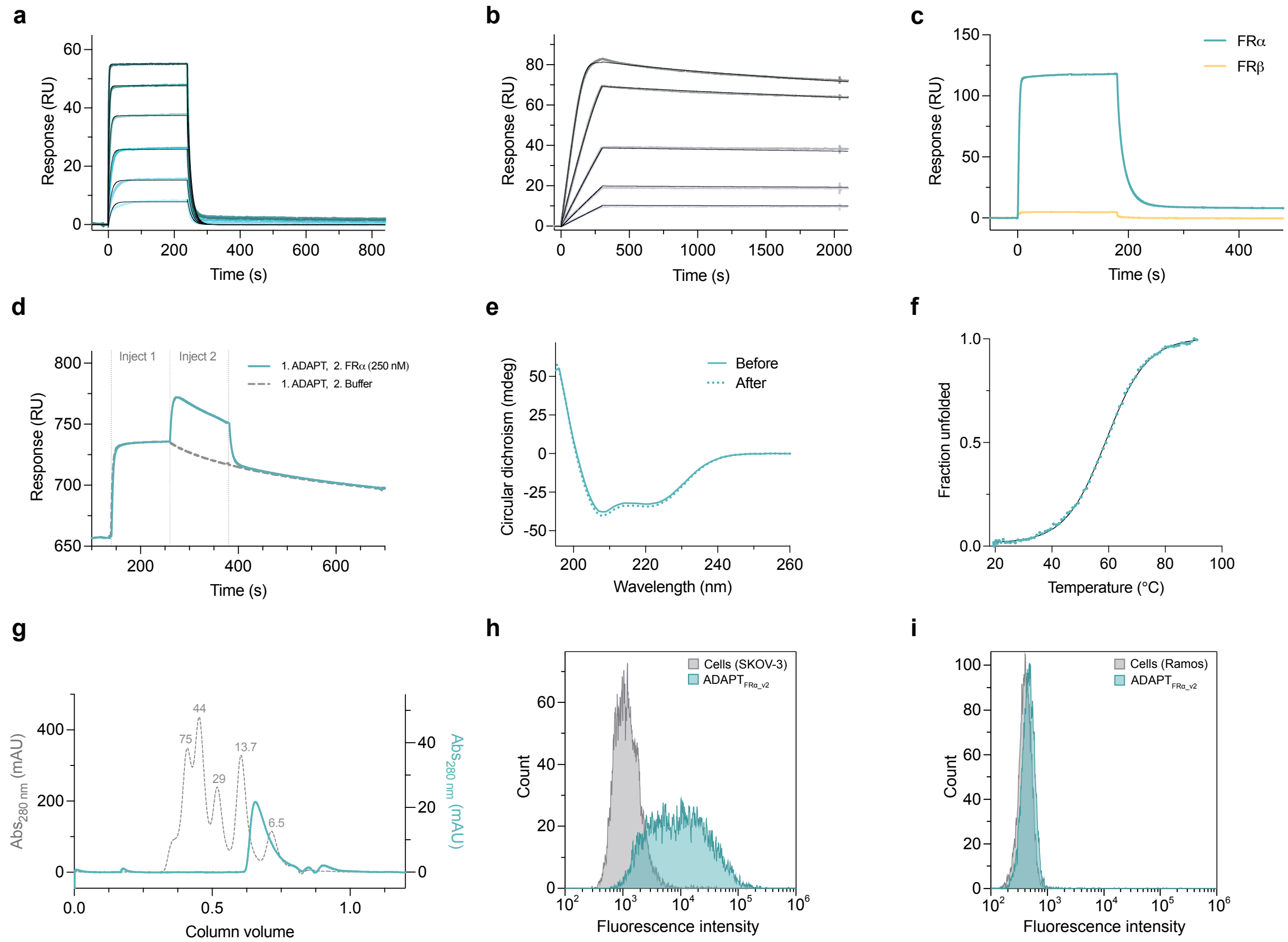

Supplementary Figure S2. Initial characterization of ADAPT$_{FR\alpha_v2}$. **a-d)** SPR evaluation of ADAPT$_{FR\alpha_v2}$ to its target proteins. **a)** A 1:2 serial dilution of ADAPT$_{FR\alpha_v2}$ was injected over immobilized FRα (443 RU) in concentrations ranging from 250 to 7.8 nM, **b)** and over immobilized HSA (1152 RU) in concentrations ranging from 3.1 to 0.20 nM. **c)** A single concentration of ADAPT$_{FR\alpha_v2}$ (250 nM) over immobilized FRα (886 RU) and FRβ (705 RU) show specificity to the alpha isoform. **d)** A first injection of ADAPT$_{FR\alpha_v2}$ (250 nM) over immobilized HSA (964 RU), followed by a second injection of FRα (250 nM), demonstrates simultaneous binding to both target proteins. **e)** CD spectroscopy of ADAPT$_{FR\alpha_v2}$ showing an alpha helical structure before and after heat denaturation, and **f)** its corresponding melting curve over 20-95°C. **g)** SEC analysis of ADAPT$_{FR\alpha_v2}$ (turquoise) confirming a monomeric nature of the binder. A calibrant (dashed grey) is included for size comparison. **h)** Flow cytometry of ADAPT$_{FR\alpha_v2}$ (1000 nM) showing binding to the ovarian cancer cell line SKOV-3 (moderate FRα-expression), **i)** and no binding to the negative cell line (Ramos).

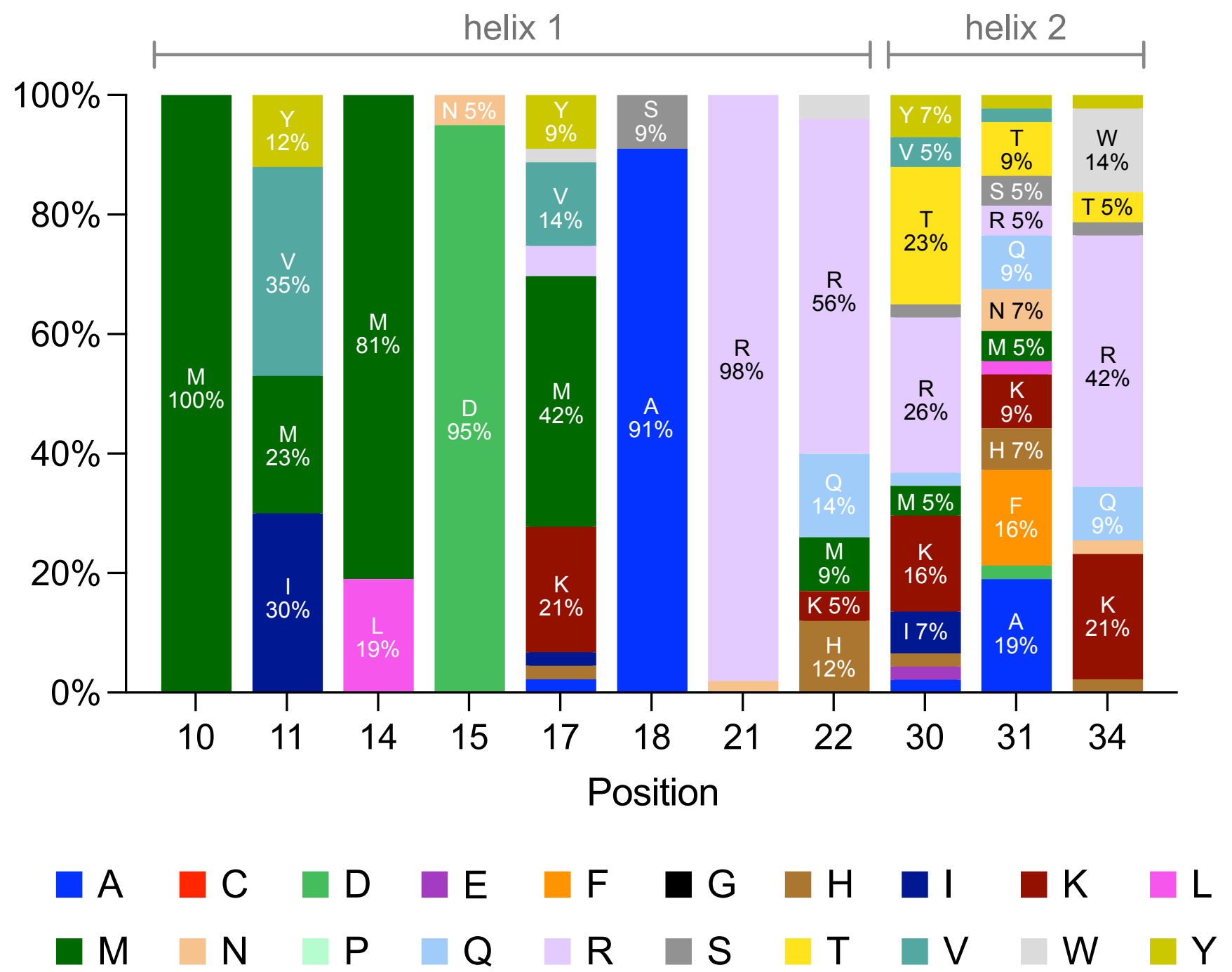

Supplementary Figure S3. The amino acid distribution in the library positions of the purple sequence cluster shown in Supplementary Fig. S1.

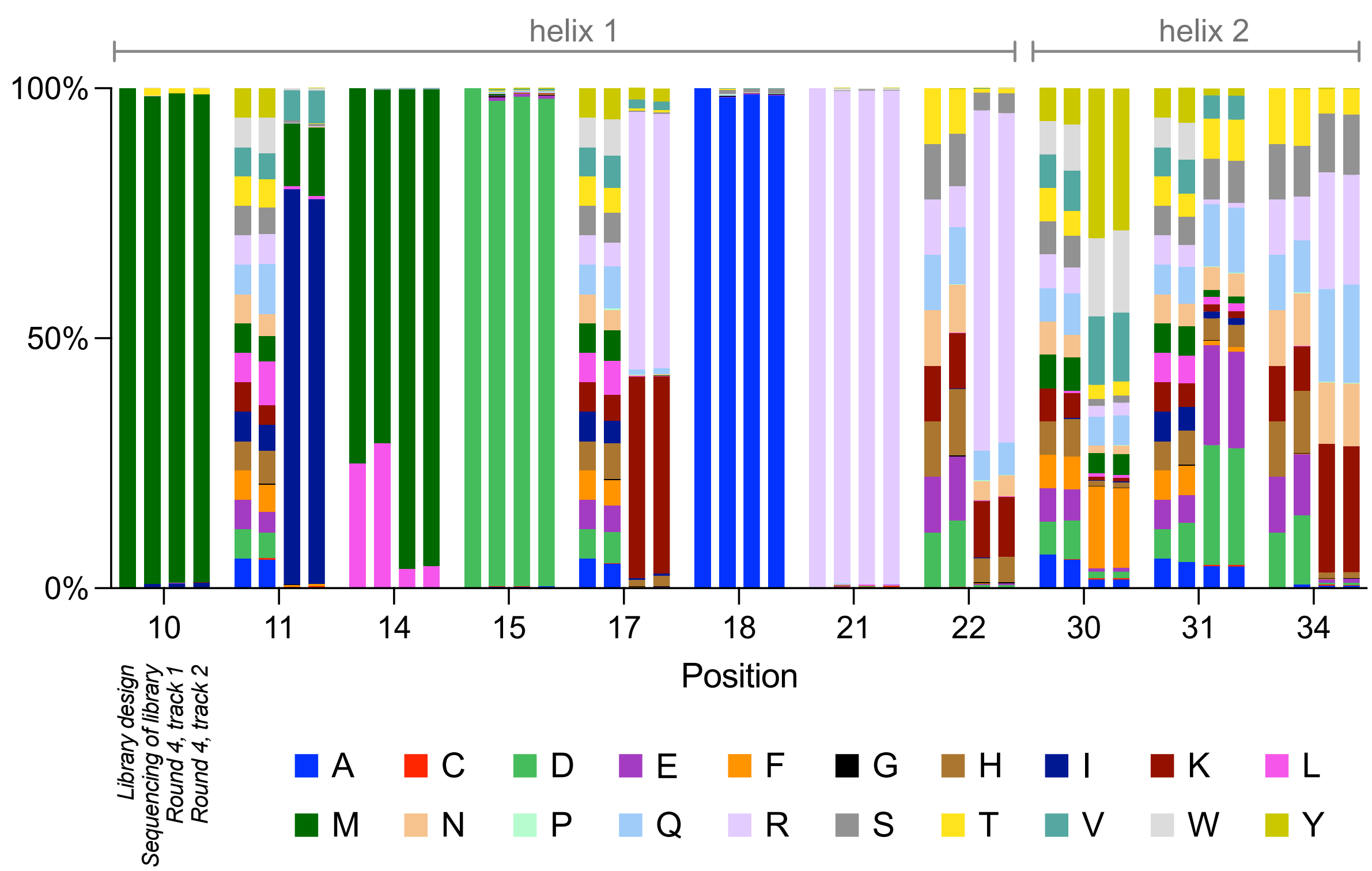

Supplementary Figure S4. The design of the first affinity maturation library compared to the deep sequencing results of the actual library and the selection output from the final panning round.

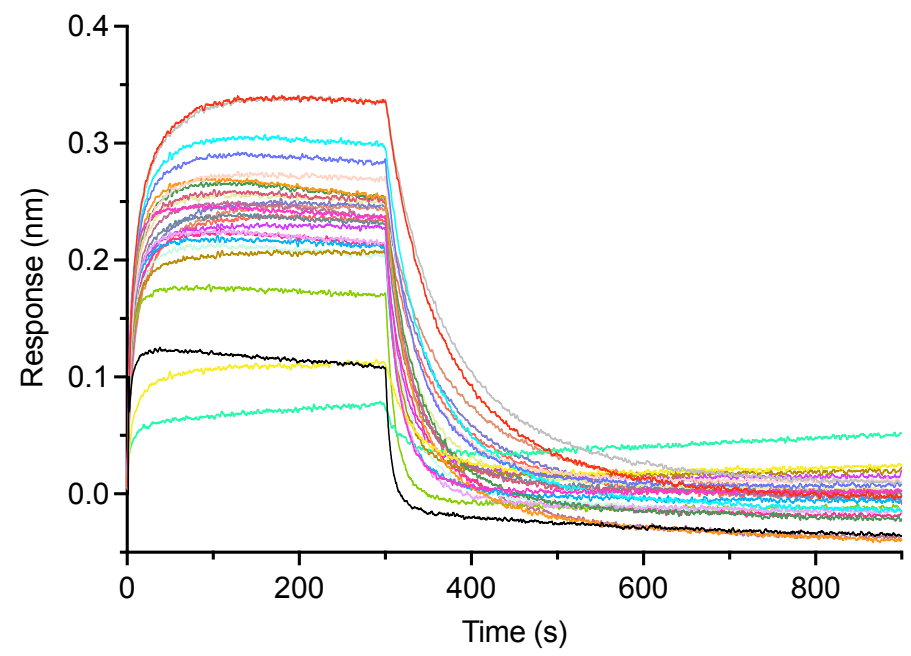

Supplementary Figure S5. Binding screen to FRα showing 24 ADAPT variants from the first affinity maturation selection. The naive binder, ADAPT$_{FR\alpha_v2}$ (black), is included for comparison. BLI screening was performed by capturing the His-tagged ADAPTs on anti-His biosensors directly from cell supernatant (loading signal not included in the figure), followed by association to 1000 nM FRα (starting at time 0), and dissociation in buffer. ADAPT$_{FR\alpha_mat1}$ is shown in red.

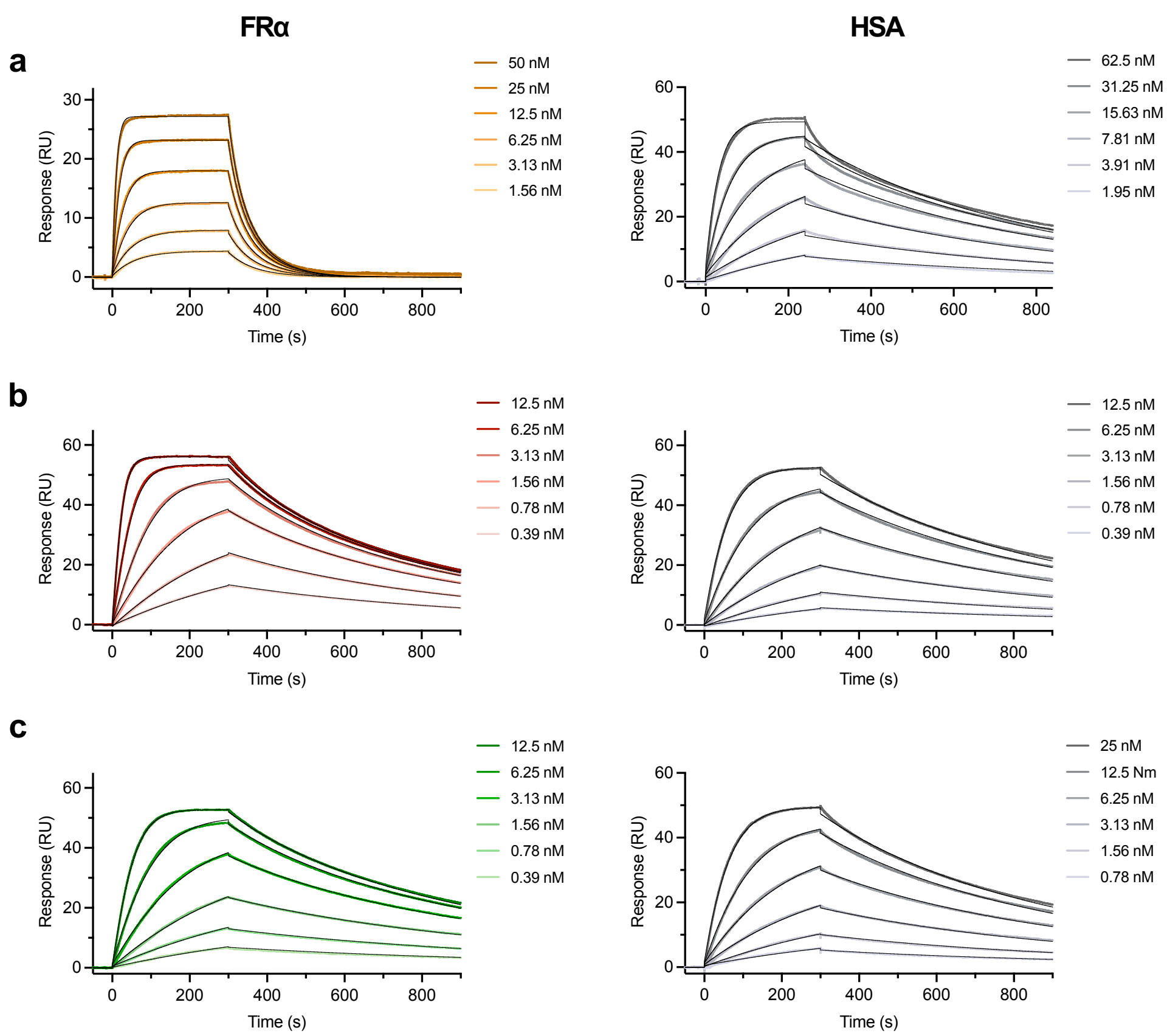

Supplementary Figure S6. Multi-cycle kinetic analysis of the affinity-matured binders in SPR. Varying concentrations of **a)** ADAPT$_{FR\alpha_mat1}$, **b)** ADAPT$_{FR\alpha_mat2}$, and **c)** ADAPT22 injected over immobilized FRα (left) or immobilized HSA (right). A 1:1 binding model was used to fit the data and compute the kinetics parameters displayed in Table 1. The fits are displayed as thin lines (black) on top of the colored data.

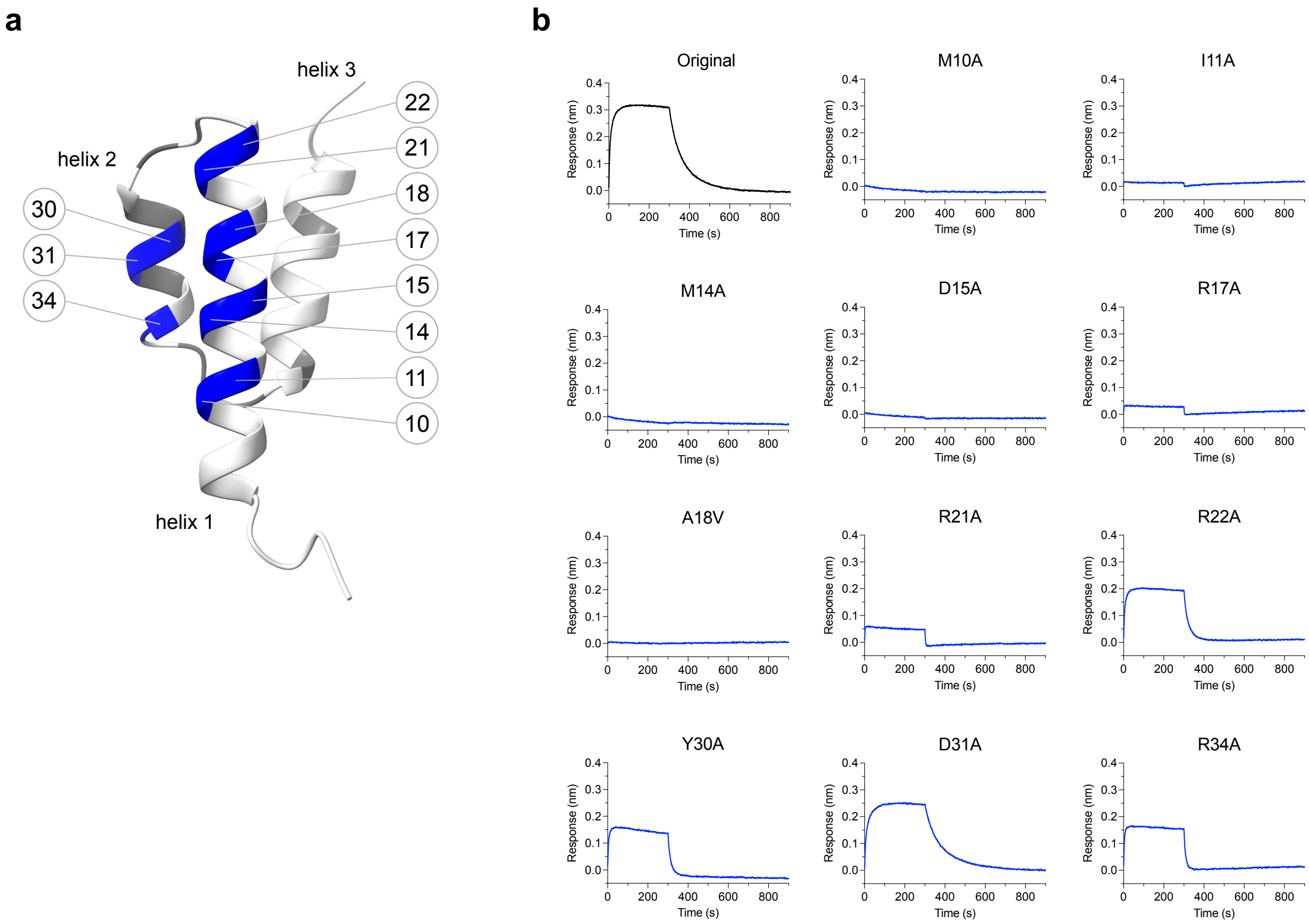

Supplementary Figure S7. Alanine scanning mutagenesis of ADAPT$_{FR\alpha_mat1}$. **a)** Schematic structure of the ADAPT binder showing the library positions (blue) which were mutated one-by-one to alanine (or to valine for position 18). **b)** BLI binding analysis of biosensor-captured ADAPT mutants (blue) to FRα in solution (1000 nM), in comparison to the original ADAPT$_{FR\alpha_mat1}$ binder (black).

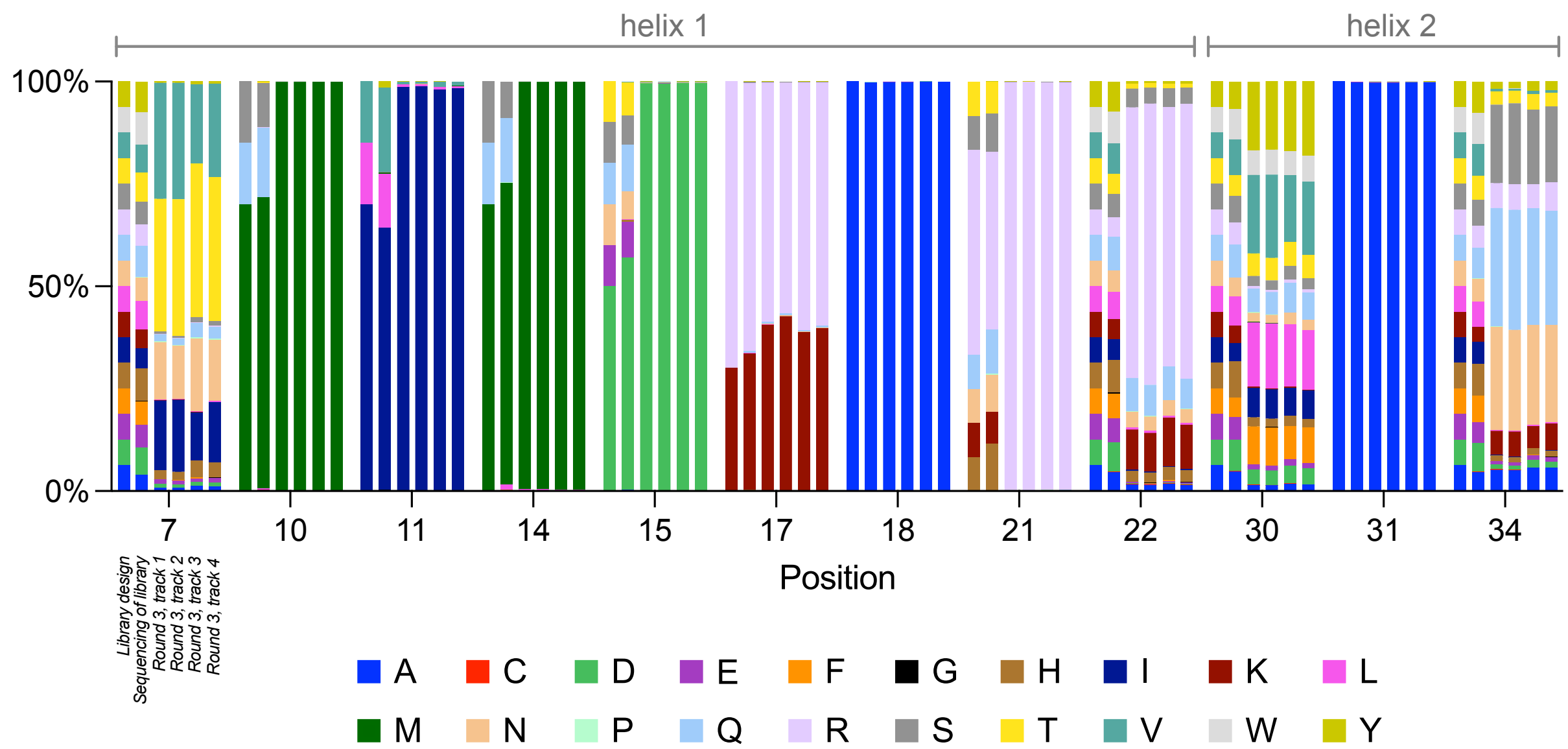

Supplementary Figure S8. The design of the second affinity maturation library together with the deep sequencing data of the experimental library and the selection output from round 3.

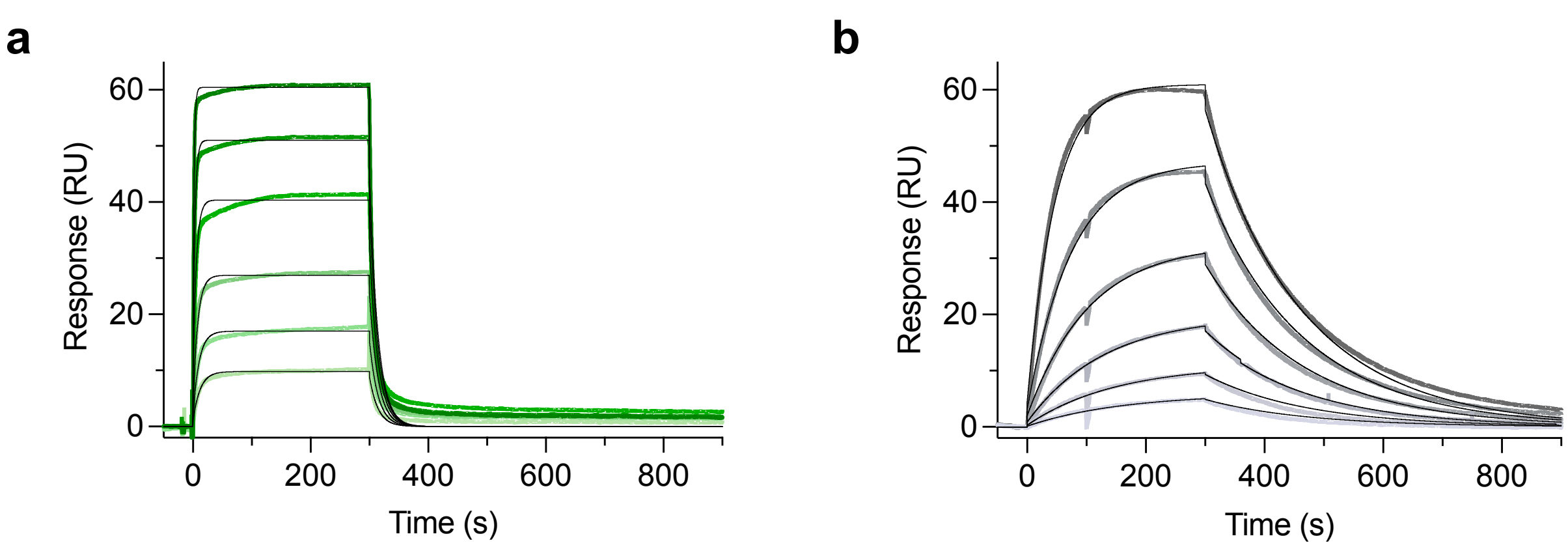

Supplementary Figure S9. SPR analysis of ADAPT22 against murine versions of the targets to check for cross-species reactivity. **a)** A 1:2 concentration series of 500 – 15.6 nM of ADAPT22 was injected over immobilized mouse FRα (496 RU). **b)** 25 – 0.78 nM of ADAPT22 was injected over immobilized MSA (1039 RU). The binding kinetics is faster to the murine targets compared to the human versions. A 1:1 binding model was used to fit the data and compute the affinity (K_D) to 77 nM (mFRα) and 9.9 nM (MSA).

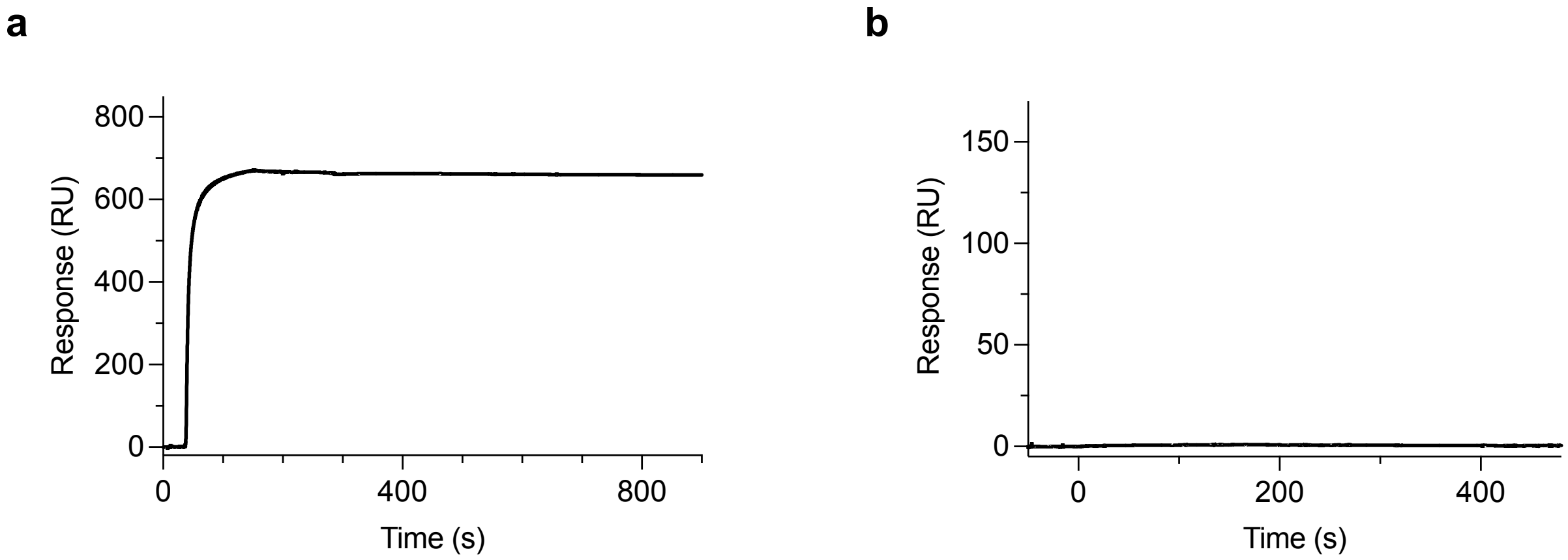

Supplementary Figure S10. SPR analysis of mirvetuximab (500 nM) injected over **a)** immobilized human FRα (451 RU) or **b)** immobilized mouse FRα (496 RU).

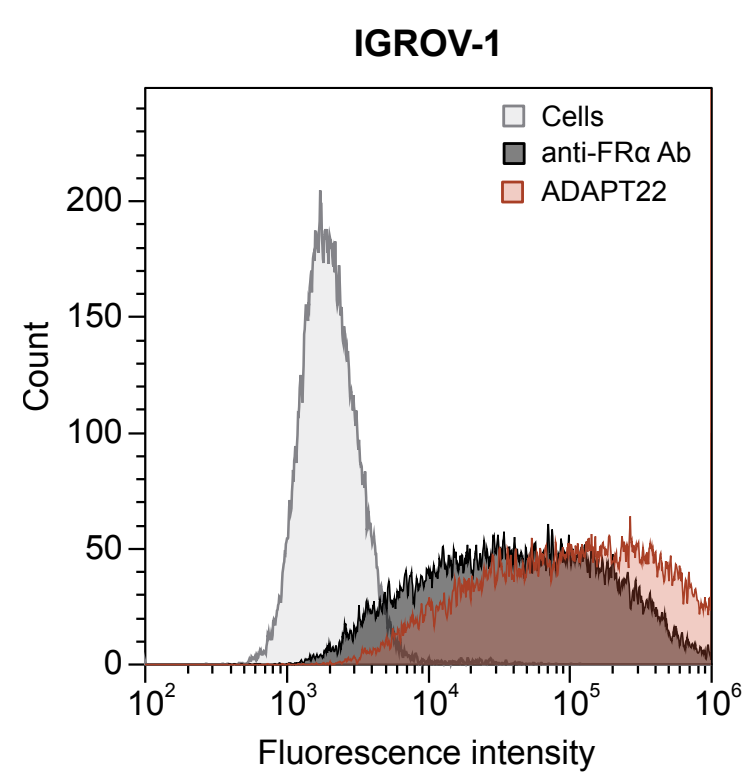

Supplementary Figure S11. Flow cytometry showing binding of 10 nM ADAPT22 (red) to IGROV-1 cells (high FRα-expression). FRα expression was verified by a monoclonal anti-FRα antibody (black).

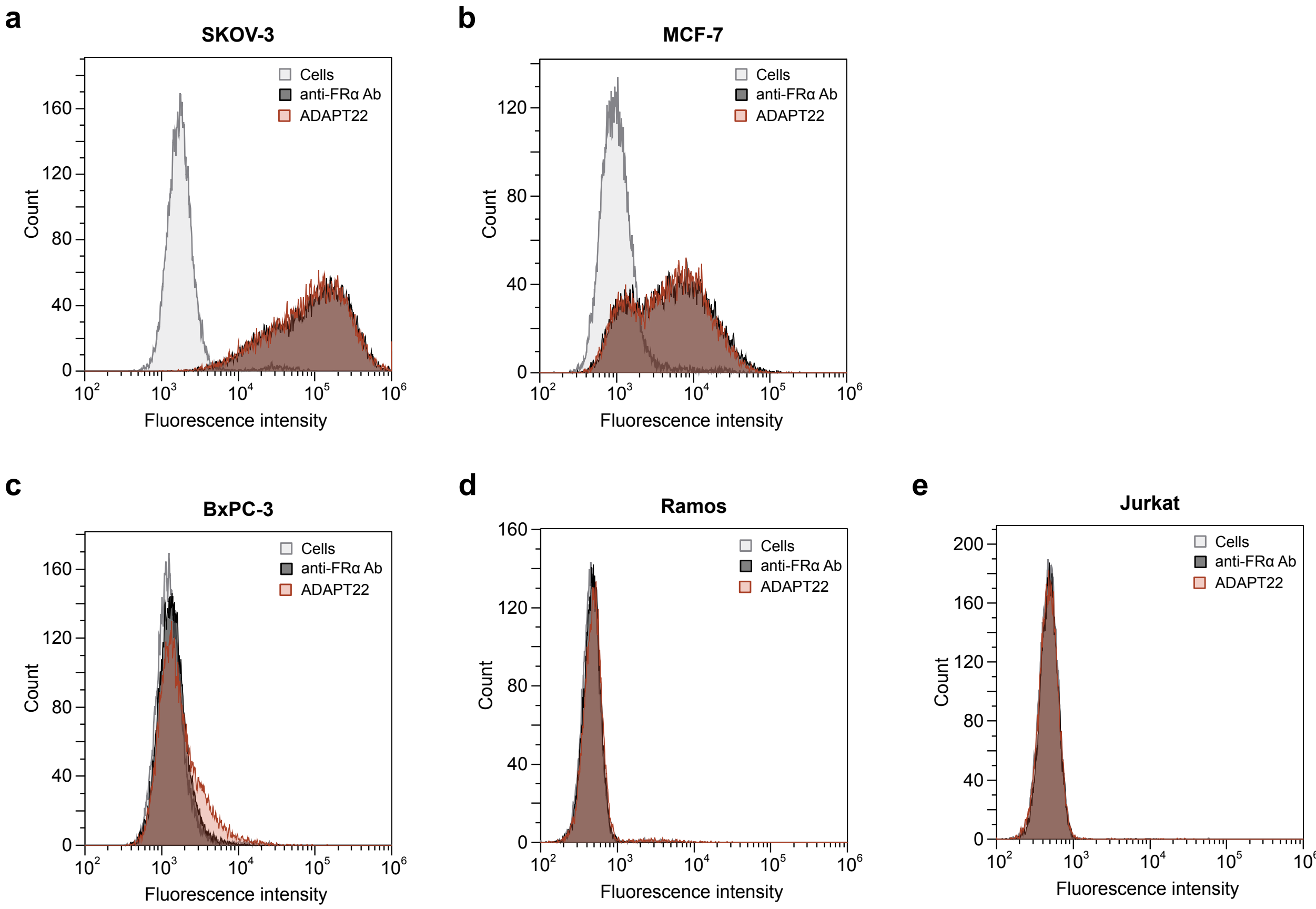

Supplementary Figure S12. Flow cytometry of ADAPT22 to various cancer cell lines. The binding of 5 nM ADAPT22 (red) was evaluated to **a)** SKOV-3 (moderate FRα-expression), **b)** MCF-7 (low FRα-expression) **c)** BxPC-3 (negative), **d)** Ramos (negative), and **e)** Jurkat (negative) cells. A monoclonal anti-FRα antibody (black) was included to confirm FRα expression.

Supplementary Table S3. Radiochemical yield (RCY), radiochemical purity (RCP), and stability of [^{99m}Tc]Tc(CO)$_3$-ADAPT22. For the stability test, the data was normalized to the value of starting RCP, which was set to 100%.

RCY before histidine (%)	RCY after histidine (%)	RCP (%)	Label stability (%)	
			Histidine (500x)	PBS (control)
96 ± 2 (n=8)	94 ± 2 (n=8)	97 ± 1 (n=8)	99 ± 0 (n=2)	100 ± 0 (n=2)

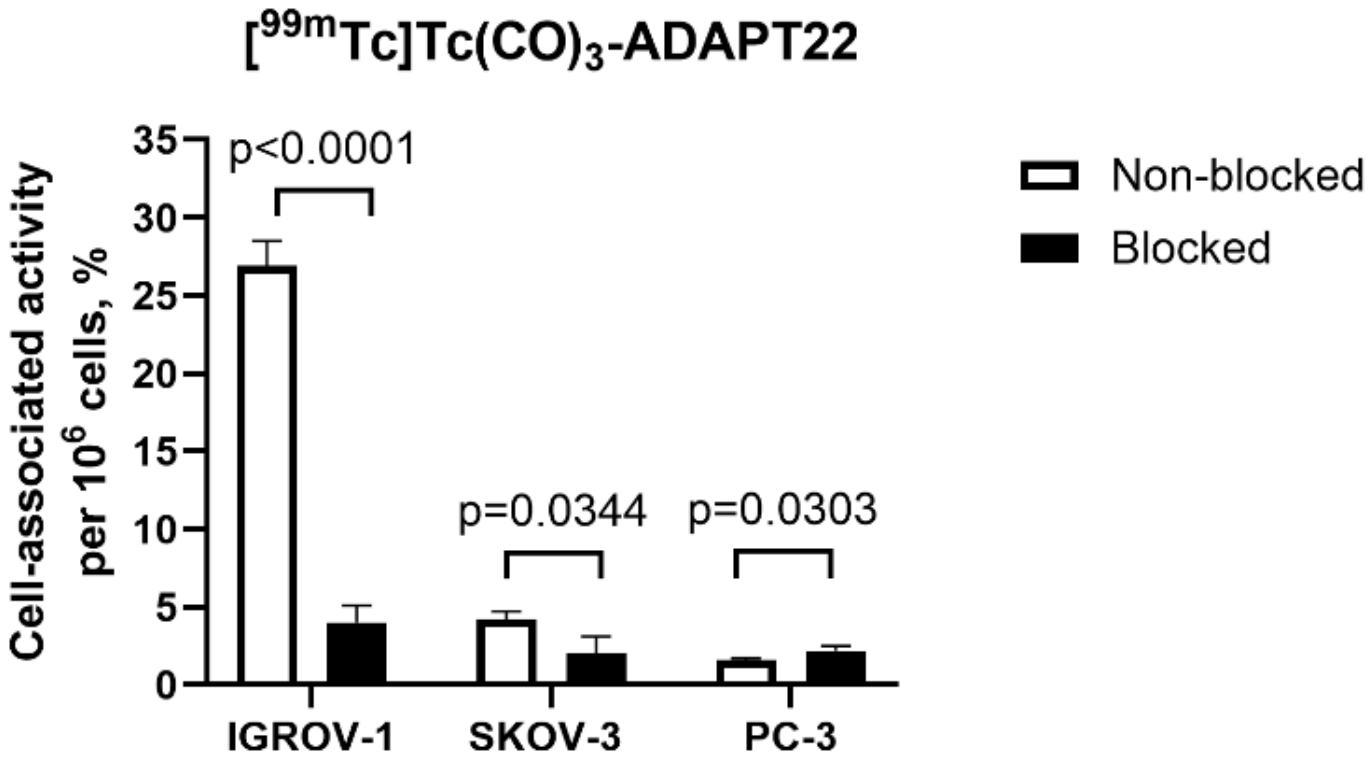

Supplementary Figure S13. FRα-binding specificity of $[^{99m}Tc]Tc(CO)_3$-ADAPT22 to FRα-expressing IGROV-1 and SKOV-3, and FRα-negative PC-3 cells. In the blocked groups, the cells were pre-incubated with unlabeled ADAPT22 (400 nM) before incubation with $[^{99m}Tc]Tc(CO)_3$-ADAPT22 (4 nM). The values are an average of three samples ±1 SD. Cell-associated activity in each sample was normalized to cell number. The experiment was performed in the presence of 100 nM HSA in cell medium.

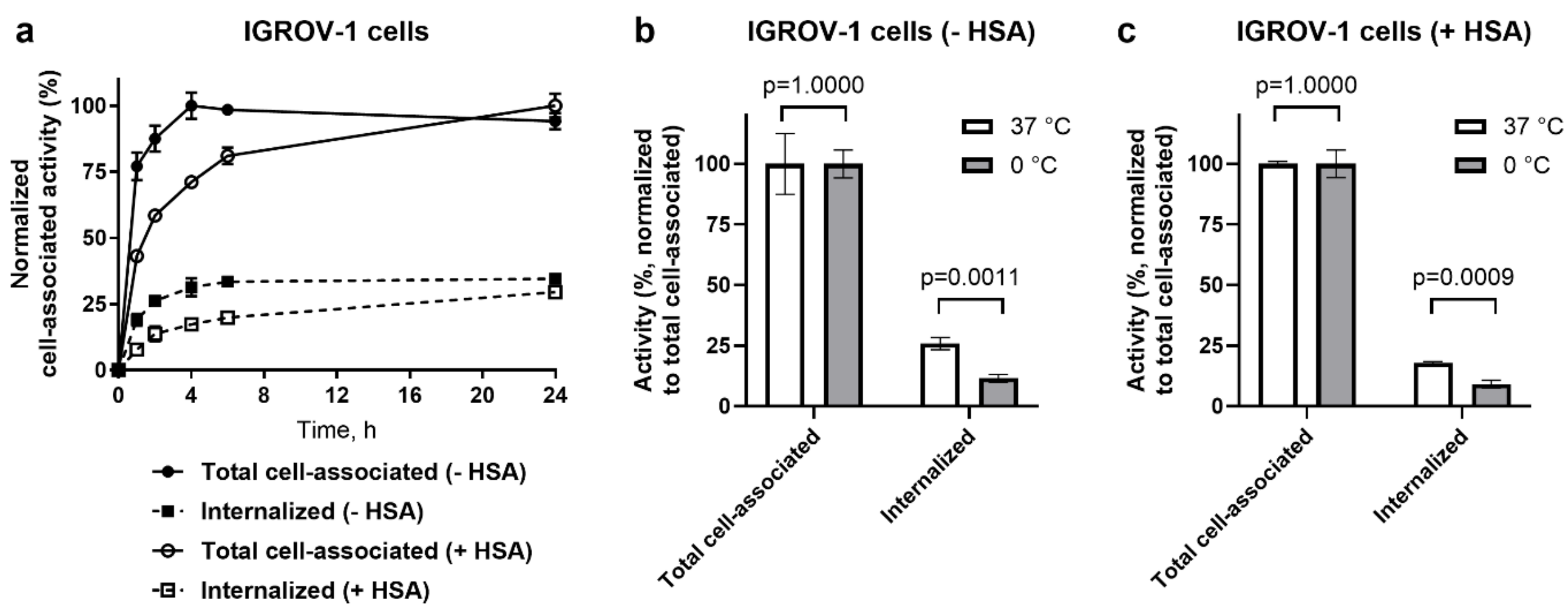

Supplementary Figure S14. Binding and internalization of $[^{99m}Tc]Tc(CO)_3$-ADAPT22 in FRα-expressing IGROV-1 cells. **a)** Continuous incubation for 24 h in the absence or presence of 100 nM HSA. Data are shown as the average ± 1 SD and are normalized to the maximal value of cell-associated activity (100%) at 24 h. **b)** Internalization after 1 h of incubation at 37 °C and 0 °C in the absence of HSA. **c)** Internalization after 1 h of incubation at 37 °C and 0 °C in the presence of HSA. The data was normalized to the maximal value of cell-associated activity taken as 100%, and analyzed by unpaired *t*-test.

Supplementary Table S4. Tissue-to-blood ratios of [^{99m}Tc]Tc(CO)$_3$-ADAPT22 in BALB/c nu/nu mice.

	4 h IGROV-1	24 h IGROV-1	48 h IGROV-1	24 h block IGROV-1	24 h PC-3
Salivary glands	0.20 ± 0.02	0.4 ± 0.1	0.4 ± 0.1	0.3 ± 0.1	0.35 ± 0.04
Lungs	0.4 ± 0.1	0.5 ± 0.1	0.6 ± 0.1	0.5 ± 0.1	0.6 ± 0.2
Liver	0.6 ± 0.1	0.7 ± 0.1	1.0 ± 0.2	0.9 ± 0.2	0.8 ± 0.1
Spleen	0.3 ± 0.1	0.50 ± 0.02	0.6 ± 0.1	0.5 ± 0.1	0.5 ± 0.1
Pancreas	0.11 ± 0.03	0.13 ± 0.02	0.13 ± 0.01	0.12 ± 0.04	0.11 ± 0.02
Stomach	0.09 ± 0.01	0.11 ± 0.02	0.11 ± 0.01	0.10 ± 0.02	0.12 ± 0.01
Small intestine	0.15 ± 0.04	0.17 ± 0.02	0.18 ± 0.02	0.15 ± 0.03	0.19 ± 0.03
Large intestine	0.11 ± 0.01	0.13 ± 0.02	0.13 ± 0.01	0.12 ± 0.02	0.13 ± 0.01
Kidneys	0.8 ± 0.1	2.6 ± 0.3	3.9 ± 0.3	3.2 ± 0.8	2.5 ± 0.4
Muscle	0.06 ± 0.01	0.10 ± 0.01	0.15 ± 0.03	0.11 ± 0.02	0.11 ± 0.02
Bone	0.10 ± 0.01	0.15 ± 0.01	0.19 ± 0.03	0.16 ± 0.02	0.17 ± 0.02
Tumor	0.6 ± 0.1	1.8 ± 0.4	2.7 ± 0.5	0.6 ± 0.1	0.5 ± 0.1
Ovaries	0.3 ± 0.1	0.4 ± 0.1	0.7 ± 0.3	0.4 ± 0.1	0.4 ± 0.1
Skin	0.20 ± 0.03	0.4 ± 0.1	0.5 ± 0.1	0.38 ± 0.03	0.4 ± 0.1
Heart	0.3 ± 0.1	0.4 ± 0.1	0.41 ± 0.04	0.4 ± 0.1	0.4 ± 0.1
Brain	0.04 ± 0.01	0.04 ± 0.02	0.04 ± 0.01	0.03 ± 0.01	0.03 ± 0.01
GI	0.2 ± 0.1	0.14 ± 0.02	0.20 ± 0.02	0.2 ± 0.1	0.21 ± 0.03
Carcass	1.5 ± 0.2	2.3 ± 0.1	2.8 ± 0.2	2.4 ± 0.4	2.6 ± 0.1

Supplementary Table S5. Tissue-to-blood ratios of [^{99m}Tc]Tc(CO)$_3$-ADAPT22 and [^{99m}Tc]Tc(CO)$_3$-ADAPT6 in NMRI mice.

	4 h ADAPT22	24 h ADAPT22	48 h ADAPT22	1 h ADAPT6	4 h ADAPT6	24 h ADAPT6
Lungs	0.4 ± 0.1	0.5 ± 0.1	0.61 ± 0.02	0.9 ± 0.1	1.3 ± 0.3	1.9 ± 0.3
Liver	0.9 ± 0.1	1.2 ± 0.2	1.4 ± 0.1	1.7 ± 0.2	5.6 ± 1.4	11 ± 1
Spleen	0.3 ± 0.1	0.5 ± 0.1	0.7 ± 0.1	0.5 ± 0.2	1.7 ± 0.3	2.6 ± 0.4
Kidneys	0.8 ± 0.1	2.5 ± 0.5	4.0 ± 0.5	374 ± 127	1194 ± 363	1859 ± 269
Muscle	0.056 ± 0.002	0.13 ± 0.02	0.16 ± 0.01	0.3 ± 0.1	0.3 ± 0.1	0.5 ± 0.2
Bone	0.10 ± 0.01	0.18 ± 0.03	0.23 ± 0.01	0.5 ± 0.1	0.7 ± 0.3	1.2 ± 0.1
GI	0.4 ± 0.1	0.7 ± 0.1	0.7 ± 0.2	2 ± 1	6 ± 4	15 ± 5
Carcass	2.3 ± 0.2	5 ± 1	6 ± 1	9 ± 2	12 ± 7	15 ± 1

Supplementary Table S6. Comparison of kidney uptake of ADAPT6 labeled with indium-111, iodine-125 and technetium-99m tricarbonyl in mice.

	Kidney uptake (%ID/g) 1 h	Kidney uptake (%ID/g) 4 h	Kidney uptake (%ID/g) 24 h
[^{111}In]In-DOTA-H$_6$-ADAPT6, NMRI mice [a]	252 ± 32	248 ± 29	225 ± 19
[^{99m}Tc]Tc(CO)$_3$-H$_6$-ADAPT6, NMRI mice [a]	286 ± 35	225 ± 12	133 ± 20
[^{99m}Tc]Tc(CO)$_3$-H$_6$-ADAPT6, BALB/c nu/nu mice [a]	306 ± 53	301 ± 58	
[^{111}In]In-DOTA-Cys^{59}-ADAPT6, BALB/c nu/nu mice [b]	272 ± 21	284 ± 21	
[^{125}I]I-HPEM-Cys^{59}-ADAPT6, BALB/c nu/nu mice [b]	10 ± 2	1.7 ± 0.3	

[a] data from Lindbo S, Garousi J, Åstrand M, Honarvar H, Orlova A, Hober S, Tolmachev V. Influence of histidine-containing tags on the biodistribution of ADAPT scaffold proteins. *Bioconjug. Chem*. **2016**, *27*, 716-26.
[b] data from Lindbo S, Garousi J, Mitran B, Altai M, Buijs J, Orlova A, Hober S, Tolmachev V. Radionuclide tumor targeting using ADAPT scaffold proteins: Aspects of label positioning and residualizing properties of the label. *J. Nucl. Med*. **2018**, *59*, 93-99.

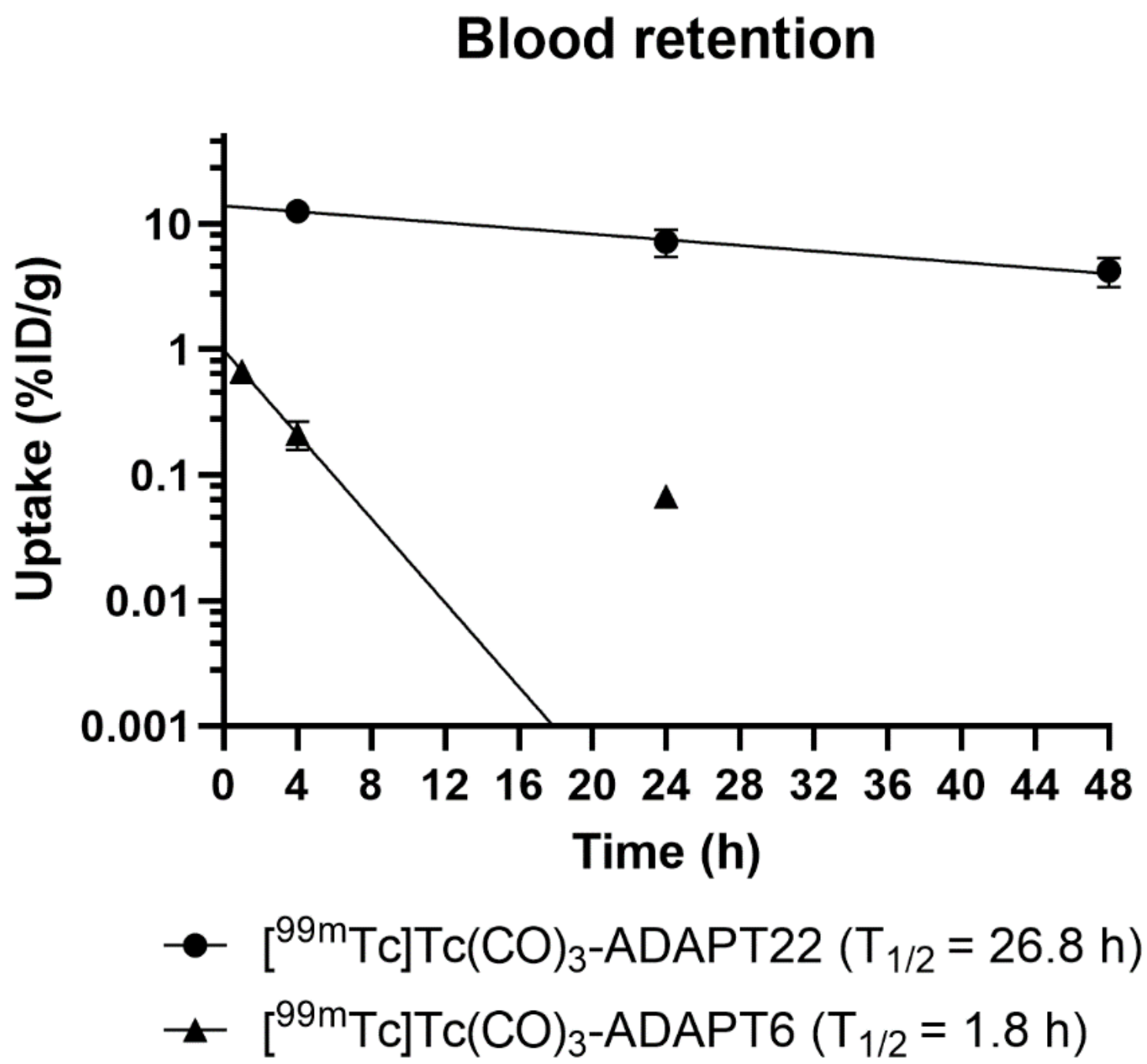

Supplementary Figure S15. Blood retention of $[^{99m}Tc]Tc(CO)_3$-ADAPT22 and $[^{99m}Tc]Tc(CO)_3$-ADAPT6 in NMRI mice plotted on a semi-logarithmic scale. The data was fitted using a one-phase decay model with the plateau fixed to zero.
